# The average individual genome: a survey of nearly 1 million whole-genome sequences

**DOI:** 10.1186/s13073-026-01792-x

**Published:** 2026-10-22

**Authors:** Harrison I. W. Wright, Robin N. Beaumont, James Fasham, Joseph S. Leslie, V. Kartik. Chundru, Timothy S. Hall, Stuart J. Cannon, Alistair T. Pagnamenta, Emma L. Baple, Andrew R. Wood, Anna Murray, Leigh Jackson, Michael N. Weedon, Kashyap Patel, Gareth Hawkes, Caroline F. Wright

**Affiliations:** Department of Clinical and Biomedical Sciences, University of Exeter Medical School, St Luke’s Campus, Magdalen Road, Exeter, EX1 2LU UK

## Abstract

**Background:**

As whole-genome sequencing (WGS) is increasingly used in clinical and public health contexts, clearer expectations regarding the amount of variation present in the “average” human genome are required to contextualise findings.

**Methods:**

We analysed WGS data from two large population cohorts: 490,541 individuals in UK Biobank (UKB) and 414,830 individuals in All of Us. Following variant and genotype QC, we aimed to assess the range of neutral and disease-associated variation across six genetically-inferred ancestry groups.

**Results:**

The “average” genome contains ~4.3–5.3 million variants, of which ~1.5% are rare (within-cohort allele frequency < 1%). This includes ~67 autosomal deletions/duplications > 10,000 basepairs per genome, and rare autosomal copy number variants > 1 Mb present in ~ 1 in 41 (2.5%) individuals. On average, ~32–42% of variants per person are homozygous and individuals have ~4 unique runs of homozygosity > 1.5 Mb, with marked variation observed across ancestry groups. Of the ~21– 26,000 SNVs/indels per person in coding exons of MANE Select transcripts, ~48% are predicted to alter the protein, of which ~0.8% are high-confidence loss-of-function and ~0.1% have REVEL score > 0.7 suggestive of pathogenicity. Depending upon parental age, the average genome contains ~63 *de novo *variants, of which ~75–80% are paternal in origin. Considering only rare variants in GenCC-curated monogenic disease-associated genes, the average genome contains ~1–3 ClinVar pathogenic or likely pathogenic variants, ~2–5 high-confidence loss-of-function variants, and ~50 ClinVar variants of uncertain significance. Most ClinVar pathogenic variants are heterozygous in recessive disease genes, consistent with carrier status, and have multiple ClinVar submitters. Finally, applying polygenic scores (PGS) for 28 common diseases within UKB, we estimate that the average person is in the top 10% for ~3 conditions, and around 1 in 5 people are in the top 1% for at least one PGS.

**Conclusions:**

The ubiquity of genetic variation in individuals has important implications for policy-makers considering genomic screening and clinicians counselling families. The continued underrepresentation of many global populations reflects a broader limitation of contemporary genomic research, underscoring the need for more inclusive datasets worldwide in order to enable equitable precision medicine.

**Graphical Abstract:**

**Figure:**
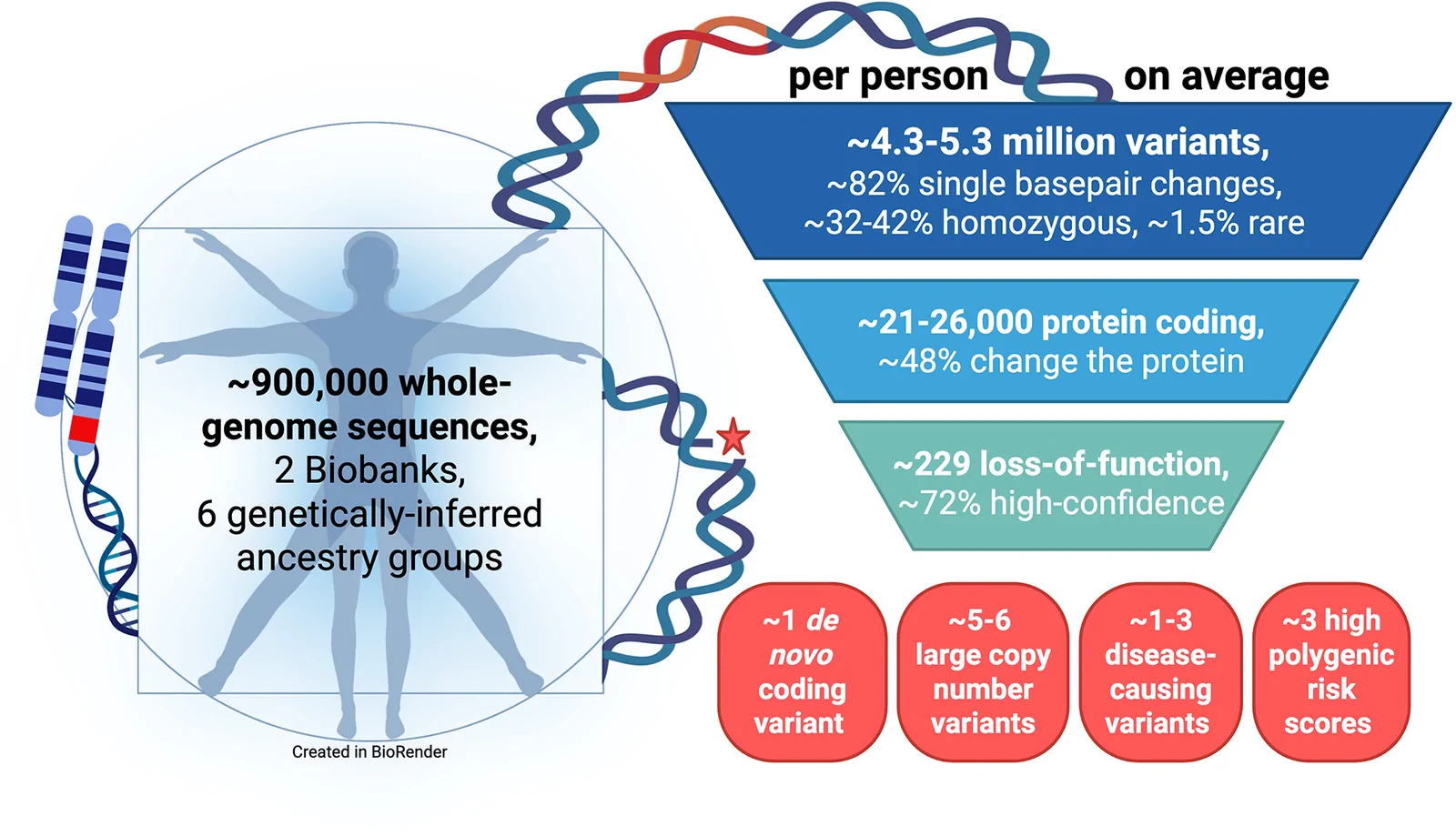

**Supplementary Information:**

The online version contains supplementary material available at https://doi.org/10.1186/s13073-026-01792-x.

## Background

Whole-genome sequencing (WGS) has now become a widespread tool in human genetics research and diagnostics [1, 2] driven by technological advancements and the falling cost of DNA sequencing [3–6]. In the 25 years since the first draft of the human genome reference sequence was released by the international Human Genome Project [7], many millions of individuals have had their exome or genome sequenced, either in a research or clinical setting, with millions more expected to be sequenced over the coming decade. WGS is also increasingly being evaluated as a tool for screening, either in newborns [8, 9] or adults [10, 11], with the hope that it can help predict and alleviate disease. Cataloguing human variation and understanding its effect on phenotypes is thus one of the foremost challenges of genomic medicine. In this context, it is important that healthcare professionals, scientists, patients and policy-makers are familiar with the extent of normal human genetic variation.

The reference human genome itself is now very well characterised. The current standard haploid human genome (GRCh38.p14) comprises ~3.1 billion basepairs (bp) across 22 autosomal chromosomes (chr) – ranging in size from ~249 million basepairs (Mb; chr1) to ~ 47 Mb (chr21) – as well as two sex chromosomes (chrX, 156 Mb; and chrY, 57 Mb) and a small circular mitochondrial genome (MT, 0.017 Mb) [12, 13]. It contains 19,871 protein-coding genes, comprising ~84 Mb of exons that are either coding (~34 Mb, ~1.2% of the genome) or untranslated region (UTR, ~50 Mb, ~1.6% of the genome), as well as 42,126 non-coding RNA genes comprising ~58 Mb (~1.9% of the genome) [14]. The rest of the genome (~95%) is non-coding and a mixture of intronic, regulatory, intragenic, repetitive, low complexity and heterochromatic regions including centromeres and telomeres.

However, the extent of genetic variation between individual human genomes has only recently become apparent. Since the first few individual genome sequences were released in 2007–9, revealing enormous numbers of novel variants [15–17], most publications have focused on cataloguing population variation within increasingly large cohorts [18]. Collaborative efforts such as the Genome Aggregation Database provide invaluable estimates of the frequency of variants in the population [19], but do not offer individual-level results. Here, we aim to provide population-based reference values for variation present in the “average” individual human genome, combining published estimates with analysis and comparison of short-read WGS data from >900,000 adults across two independent population-scale biobanks, UK Biobank [20] and All of Us [21]. Individuals in these biobanks were not selected on the basis of any particular disease, and were all adults at the point of recruitment; their genomes therefore reflect the distribution of “normal” genetic variation that might be present in individuals drawn from overlapping populations, irrespective of health status.

## Methods

### UK biobank (UKB)

The UKB ‘500k’ release includes 490,541 individuals with WGS data, recruited by invitation to one of 22 assessment centers [20, 22]. Participants ranged from 37 to 73 years of age at recruitment, and 54% of participants were female (Figure S1 and Table S1). WGS was performed on blood-extracted DNA using short-read Illumina NovaSeq 6000 sequencing technology with an average coverage of 32.5x [20]. Sequence reads were mapped to Genome Reference Consortium Human build 38 (GRCh38); single nucleotide variants (SNVs) and short (<50bp) insertion/deletions (indels) were jointly called using DRAGEN 3.7.8 [23]. We used bcftools [24] to apply genotype-level filters (GQ > 10, DP > 7, and missingness < 0.1) in addition to existing DRAGEN-ML variant-level filters, as detailed elsewhere [25], split multi-allelic sites, and left-aligned all REF and ALT alleles using a 1000 Genomes Project b38 reference (accessed 30/03/2024). We then assigned all variants a unique ID (CHR:POS:REF:ALT), followed by the application of plink2 [26] to convert each filtered project Variant Call Format file (VCF) shard to plink2 format, and finally concatenated into per-chromosome p(gen/sam/var) files. Single-sample VCFs of copy number variants (CNVs) generated using DRAGEN were available for 490,414 (99.9%) of individuals, and we exclusively considered autosomal CNVs > 10,000 basepairs (bp). We removed CNVs with a mean QUAL score across all carriers ≤ 95th centile of the means (17 and 19 for deletions, DELs, and duplications, DUPs, respectively). For the mitochondrial (MT) genome, variants were called using mitoHPC [27]; 485,502 individuals (99%) and 16,446 MT variants (98%) passed QC and were included [28].

The majority of individuals (95.7%) were grouped into five genetically-inferred ancestry groups, based on similarity with 1000 genomes project (1KG) clusters [29]: European (UKB-EUR; *N* = 448,608; 91.5%), African (UKB-AFR; *N* = 7,901, 1.6%), South-Asian (UKB-SAS; *N* = 10,129; 2.1%), East-Asian (UKB-EAS; *N* = 2,543; 0.52%) and Admixed American (UKB-AMR; *N* = 466; 0.10%). Briefly, principal components (PCs) were generated in 1KG and projected into UKB participants using a set of variants that were of high quality in UKB. K-means clustering was performed on PCs 1–4 initiated using 1KG ancestry populations to define UKB ancestry groups. We used pre-calculated non-enhanced polygenic risk scores (PGSs) available through the UKB-RAP from fields 26,202 to 26,289, limited to a subset of 28 disease-related PGSs (Table S2). Percentiles were calculated per score, and the number of scores above a given centile were calculated for each individual.

### All of us (AoU)

The AoU v8 release includes 414,830 individuals with WGS data, recruited through a network of more than 340 sites, including health care providers, community partners and online registration [30]. Participants ranged from 18 to over 90 years of age at biosample collection, and 61% of participants were female (Figure S1 and Table S1). WGS was performed on either blood-extracted DNA (*N* = 365,931) or saliva-extracted DNA (*N* = 48,899) using short-read Illumina NovaSeq 6000 sequencing technology with an average coverage of 37.9x [21]. Sequence reads were mapped to GRCh38; SNVs and short indels were called using DRAGEN 3.7.8 [23]. Joint calling was performed via the Genomic Variant Store [21]. The full AoU v8 WGS dataset was provided exclusively in HAIL VDS format, with VETS filtering flags. We exported the Hail VDS to VCF format using Hail v0.2.134-952ae203dbbe. We applied genotype-level filters (GQ ≥ 20, DP > 7, and missingness < 0.1) on top of the allele-level markers provided by VETS [25]. Jointly-called structural variants (SVs) were available for 97,061 (23.4%) of individuals in AoU using GATK-SV [31, 32] aligned with DRAGEN 3.4.12 (distinct to DRAGEN 3.7.8 used for joint calling of SNVs and indels), with additional QC [33]. For parity with UKB, we only considered autosomal DUP and DEL-type SVs ≥ 10,000 bp in length for our analysis of CNVs and removed those with a QUAL score ≤ 95th centile (67 and 41 for DELs and DUPs, respectively).

All individuals were grouped by AoU into six previously defined genetically-inferred ancestry groups, as described elsewhere [30, 34]: European (AoU-EUR; *N* = 234,353; 56.5%), African-American (AoU-AFR; *N* = 84,148; 20.3%), Admixed-American (AoU-AMR; *N* = 79,106; 19.1%), Middle Eastern (AoU-MID; *N* = 1,545; 0.37%), South Asian (AoU–SAS; *N* = 5,579; 1.35%) and East Asian (AoU-EAS; *N* = 10,099; 2.44%).

### Annotation of variants (both cohorts)

SNVs and indels were annotated with the Ensembl Variant Effect Predictor (VEP, Ensembl release 115) [35], LOFTEE [18] and REVEL [36]. Lists of variants with consequences of interest (genome-wide worst consequences across all Gencode Primary release 49 transcripts, and coding consequences restricted to MANE Select transcripts) were then extracted and grouped following the logic in Table S3. Pathogenic (P) variants, likely pathogenic (LP) variants and variants of uncertain significance (VUS) were downloaded from ClinVar (accessed 08 December 2025) [37]. P/LP/VUS were then subsetted into monogenic disease-associated genes with modes of inheritance from Gene Curation Coalition (GenCC) (accessed 03 February 2026) [38] using genes with definitive, strong and moderate associations that had not been refuted or disputed (Table S4). CNVs were annotated against Genome in a Bottle (GIAB) problematic regions [39] using bedtools [40] and GATK-SVannotate [31, 32]. In UKB, we annotated CNVs based on Gencode Primary transcripts (v49) [41], whilst in AoU we used the CNV annotations provided.

### Calculation of variant frequencies and counts

Minor allele frequencies (MAFs) were calculated both per-cohort and per-genetically-inferred-ancestry-group within each cohort, using plink2 with the option ‘--freq counts cols = altfreq,nobs’. Per-sample statistics were generated by subsetting to the desired list of variants (using --extract) and the option ‘--sample-counts zs cols=“homref,homalt,het”’, with additional cols haprefwfemaley and hapaltwfemaley on chromosome X. Per-chromosome and per-variant-list sample statistics were merged within each cohort via a custom script using the Polars Python module. In order to plot and summarise results across both biobanks, per-sample variant counts were binned across samples and variant categories, with a minimum of five samples in each bin in UKB, and the UKB and AoU bins were merged in the AoU Researcher Workbench. We excluded a total of 5,017 AoU samples when summarising per-sample results, due to one or more of the following reasons: AoU known issue 1 (data quality issue, *N* = 3); AoU known issue 10 (mean srWGS coverage < 30x, *N* = 4044); AoU flagged population outliers (*N* = 987) [33].

To represent the variation across all the genetically-inferred ancestry-similarity groups in the study, where we give ranges we calculate interquartile ranges for all groups and show the minimum of all the first quartiles and maximum of all the third quartiles across all groups. Where we give ranges for ‘1 in N’ people, we calculate the mean per group and show the minimum and maximum across all groups.

### Detection of de novo variants (both cohorts)

Parent-offspring trios were identified from the relatedness inference using KING [42] on the microarray genotype data in both cohorts. Variants were called using bcftools, and bcftools trio-dnm2 was used to assign a probability of each variant being *de novo*. Using a lenient filter of dnm > −0.3566749 (equivalent to a probability of 0.7, given the vcf tag is log scaled), candidate *de novo* variants were then input into denovoCNN [43] which also assigns a probability of the variant being a true *de novo* variant. For the analysis of constitutive variants, we removed those with a low variant allele fraction (VAF < 0.25) in the offspring and supporting alleles (VAF > 0.05) in either parent. We input these probabilities (in addition to the genotype quality and read depths of the offspring and parents, and the mapping quality of the offspring) into an optimisation algorithm [44] which chooses the thresholds for these parameters to best align with the paternal age association from a larger published dataset of *de novo* variants [45], using an initial short run to remove outliers with >200 *de novo* variants (three individuals from each cohort were removed due to outlying numbers of variants). We removed variants in low complexity regions, and known segmental duplication regions defined by GIAB [39]. Once the thresholds were set, we applied the same thresholds to candidate *de novo* variants with offspring VAF < 0.25 to obtain probable mosaic *de novo* variants, while removing individuals with outlying numbers of variants (9 individuals were removed from both cohorts).

### Detection of runs of homozygosity (both cohorts)

Per-individual runs of homozygosity (ROHs) were estimated using a Hidden Markov Model implemented in bcftools v1.22 [24]. We exclusively considered biallelic single-nucleotide variation with MAF ≥ 1%, excluding GIAB problematic regions [39], and subsequently applied LD-pruning within each genetically-inferred ancestry group using plink2 (with flags --maf 0.01 --indep-pairwise 50 5 0.8). We then filtered to ROHs with a PHRED quality score ≥ 20.

## Results

### The average individual genome has ~4.3–5.3 million variants compared to the current reference

**Fig. 1:**
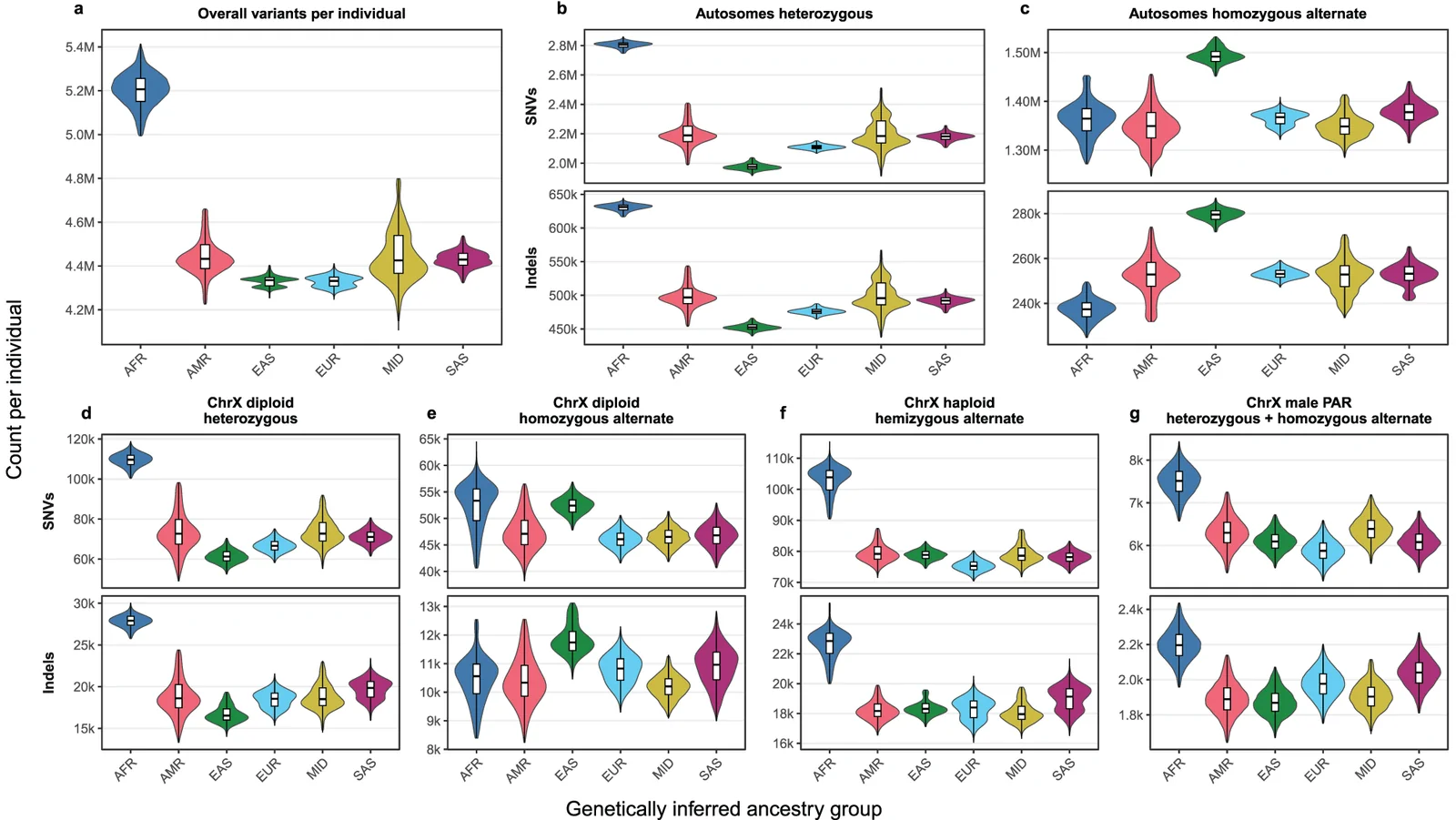
Number of SNVs and indels per individual in UKB and AoU compared with the GRCh38.p14 reference genome. Median and interquartile ranges shown, violins clipped to range of whiskers (1.5x IQR). **a**) overall number of variants per individual; **b**-**g**) number of SNVs (top) and indels (bottom) per individual by category: **b**) heterozygous autosomal variants, **c**) homozygous alternate autosomal variants, **d**) heterozygous variants on chromosome X in females, **e**) homozygous alternate variants on chromosome X in females, **f**) hemizygous alternate variants on chromosome X in males **g**), combined heterozygous and homozygous alternate variants in the pseudo-autosomal region (PAR) in males; AFR = African (dark blue), AMR = Admixed American (pink), EAS = East Asian (green), EUR = European (light blue), MID = Middle-Eastern (yellow), SAS = South Asian (purple)

Using WGS data from 879,461 adults combined across UKB and AoU, we found that the typical genome contains ~4.3–5.3 million SNVs and short indels (< 50 bp), with the most variation seen in individuals genetically similar to the 1KG-AFR group, consistent with numerous previous studies [18, 29, 46–48] (Fig. 1 and Table S5). Relative to other groups, the AFR-like group has ~1 million more total variants called on average, which is the result of both ~2–4 million fewer reference calls and ~1–3 million more missing calls (Figure S2 and Table S6), potentially caused by poor alignment with the current reference genome, which is derived predominantly from EUR-like individuals. On the autosomes, an average of 1.6–1.8 million (32–42%) of an individual’s variants are homozygous and 2.4–3.5 million (58–68%) are heterozygous, of which ~2% are multiallelic (i.e. heterozygous for two alternative alleles in the same individual). In addition, females have ~75–140 thousand heterozygous and ~55–67 thousand homozygous variants on chrX, whilst males have ~92–130 thousand hemizygous variants on chrX (Fig. 1), ~5.5 thousand variants on chrY [49], and ~7–10 thousand variants in the pseudoautosomal (PAR) region. Based on previous work on the mitochondrial genome [28], we found that individuals in UKB have a mean of ~3.98 heteroplasmic variants (present in 10–90% of MT genomes) and ~8.45 homoplasmic variants (shared by ≥ 90% of MT genomes), detectable in blood. This number of heteroplasmic MT variants is elevated compared with some previous literature [50, 51], which may be explained by the accumulation of age-related MT mutations [52], highlighting potential ascertainment bias as well as the impact of different QC and variant filtering methods.

Unlike small (< 50 bp) variants, the number of structural variants (SVs) detected per individual genome is highly dependent upon the technology used for genotyping – including microarrays of varying resolution and short-read versus long-read sequencing – and the algorithms used for variant calling [23, 53–55]. The majority of the differences between studies are explained by small CNVs of < 500bp, as well as short tandem repeats (STRs), inversions and retrotransposons [56], which are hard to detect with most technologies. Recent high-resolution analyses estimate a median SV count per person of 22,000–26,000 variants, dependent upon genetically-inferred ancestry groups, affecting ~5-times the genomic footprint of SNVs [47, 55, 57, 58].

**Fig. 2:**
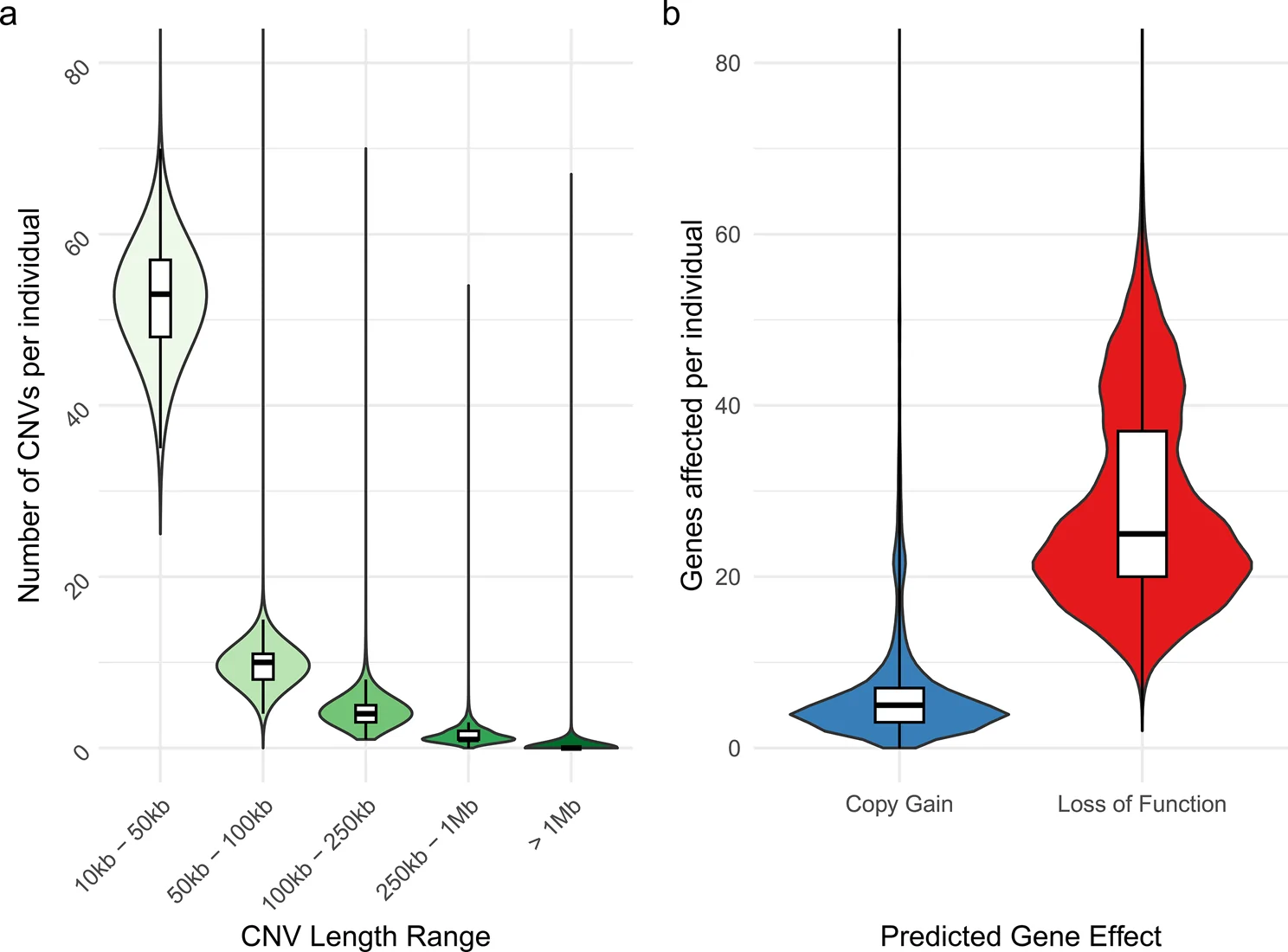
Distribution of CNVs, and genes affected, per individual compared with the GRCh38.p14 reference genome. **a**) number of CNVs carried per individual; shade of green represents the size (median and interquartile range shown, outliers removed); **b**) number of genes affected by CNVs, split by copy gain (predicted full copy gain; blue) and predicted loss-of-function (where any protein-coding sequence is lost or disrupted; red). CNV = copy number variant; kb = kilobasepairs; Mb = megabasepairs

Because most SVs are difficult to call robustly with short-read WGS data, we limited our analysis to high-quality CNV calls > 10,000 bp in length, as has been done previously [59], which were available in UKB and AoU in 587,475 individuals. Excluding CNVs overlapping Genome In A Bottle (GIAB) problematic regions by ≥ 75% (threshold chosen based on the expected vanishingly low frequency of jointly-called CNVs with length > 1Mb; Figure S3), we estimate that individuals have a median of 68 CNVs (46 deletions and 22 duplications) of > 10,000 bp (Fig. 2a, Figure S4 and Table S7), consistent with previous observations in UKB [59]. Around 1 in 159 individuals (0.63%) in UKB and AoU have a > 1 Mb deletion and 1 in 55 (1.8%) a > 1 Mb duplication, the majority of which are singletons, consistent with previous observations using microarray-based genotyping [60]. Given the age at recruitment across these two adult cohorts, with a mean of 57 years in UKB and 52 years in AoU [61], this figure may be an overestimate of germline CNVs. Age-related clonal hematopoiesis likely accounts for some large CNVs, including chromosome aneuploidies [62–64], and some STRs also expand with age [65].

Based on common variation (MAF ≥ 1%) alone, we found that the autozygosity within an individual varied by genetically-inferred ancestry group, in line with previous work [66] (Figures S5-6). On average, individuals in UKB had 3.65 unique runs of homozygosity (ROH) of length ≥ 1.5 Mb (ranging from 1 to 62 ROHs), and in AoU individuals had on average 4.00 ROHs (ranging from 1 to 73 ROHs). However, this varied substantially by genetically-inferred ancestry group; for example, AoU-SAS individuals had on average 5.27 ROHs > 1.5 Mb, whilst AoU-MID individuals had an average of 7.14. We did not assess the degree to which ROH represents hemizygosity in regions deleted by structural variation.

By including smaller ROHs, we estimated the density of homozygosity in 0.1 Mb bins for each of the autosomes (Figure S7). ROH length varied up to 242 Mb in UKB, representing almost the entirety of chromosome 2, and up to 248 Mb in AoU, representing almost the entirety of chromosome 1. We therefore further investigated individuals exhibiting near-complete homozygosity on a single chromosome, caused either by constitutive uniparental disomy (UPD) or somatic loss of heterozygosity (LOH) [67]. Based on a threshold of > 90% chromosomal homozygosity, and an assumed background of < 2-fold homozygosity on any given chromosome compared to the mean chromosomal homozygosity for that individual (excluding the most-homozygous chromosome), we found that 1 in ~8,100 people were potential cases of UPD/LOH, which is lower than anticipated for UPD [68] consistent with a healthy-volunteer bias in adult population cohorts [69].

### The majority of variants in the average human genome are common and non-coding

**Fig. 3:**
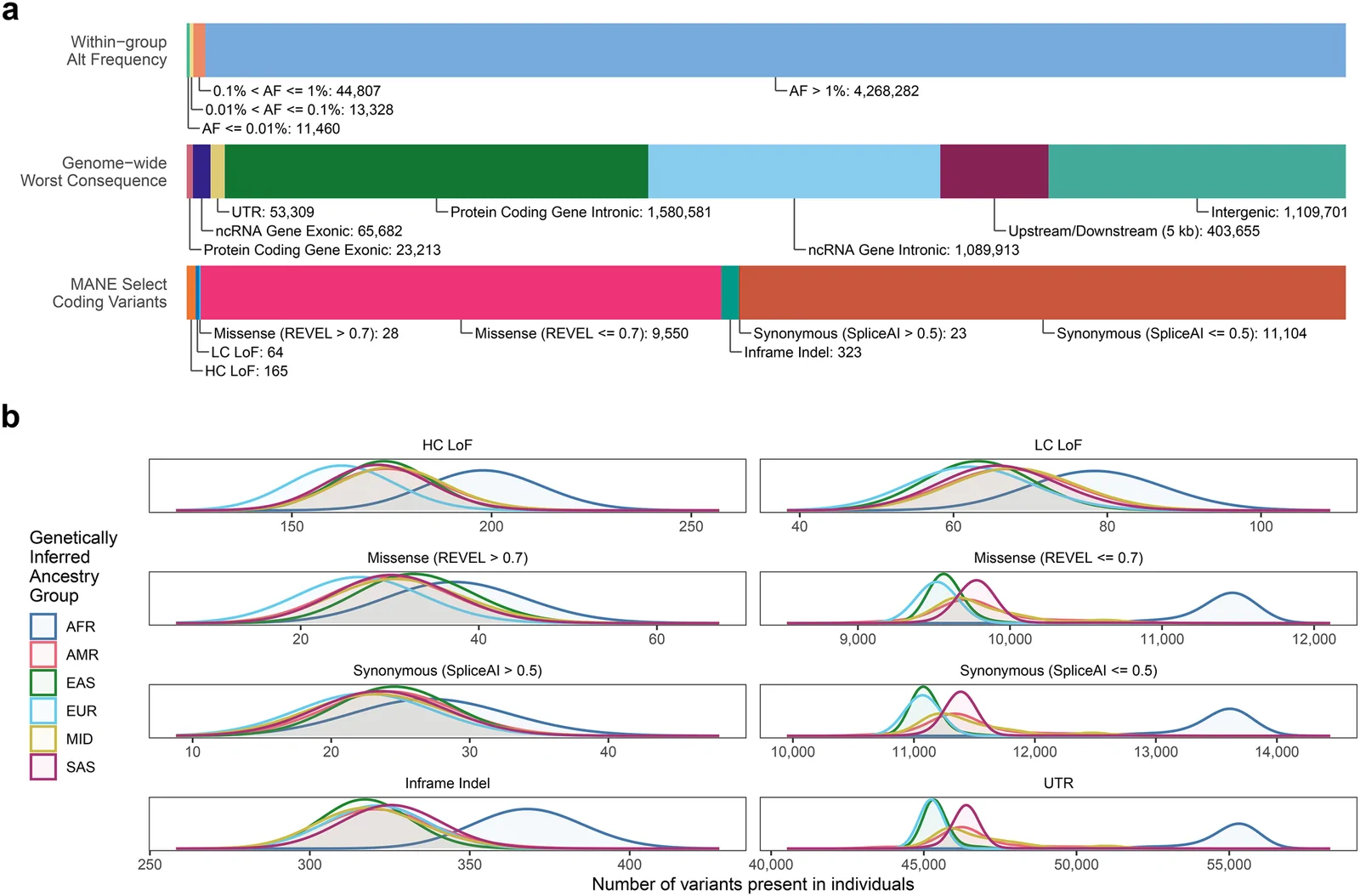
Number of SNVs and indels per individual in different frequency and consequence categories across six genetically-inferred ancestry groups. **a**) median total SNVs/indels split by allele frequency and consequence; **b**) distributions of number of variants in MANE Select transcripts across different consequences and ancestry groups. AF = allele frequency, UTR = untranslated region, ncRNA = noncoding RNA gene, LC LoF = low-confidence loss-of-function, HC LoF = high-confidence loss-of-function; AFR = African, AMR = Admixed American, EAS = East Asian, EUR = European, MID = Middle-Eastern, SAS = South Asian

Unlike genetic databases, in which the majority of variants are rare, most variants observed in the average individual genome are common. Based on within-cohort genetically-inferred ancestry group allele frequencies, a median of 98.4% of the SNVs/indels in an individual genome are common with a MAF > 1%, whilst 1.34% have MAF 0.01–1% (rare) and 0.26% have MAF < 0.01% (ultrarare, Fig. 3a). Considering the most severe consequence across all Gencode v49 transcripts [41], 38.3% of SNVs/indels occur within protein-coding genes, including 0.54% in protein-coding regions, 1.2% in UTRs and 36.5% in introns (Fig. 3a and Table S8). An additional 26.7% fall within non-protein-coding genes (1.5% exonic and 25.2% intronic), whilst the remaining 35% lie outside annotated protein or RNA genes and have no VEP-predicted functional consequence. Considering only MANE Select transcripts of protein-coding genes (*n* = 19,319) [70], the median human genome contains (Fig. 3a): 9,600 missense variants, of which 28 (0.29%) have a REVEL score > 0.7 suggesting a higher likelihood of pathogenicity [36]; 11,100 synonymous variants, of which 23 (0.21%) have a SpliceAI score > 0.5 suggesting an effect on splicing [71]; and 229 predicted loss-of-function (pLoF) variants (including stop-gain, start-loss, frameshift and splice acceptor/donor variants), of which 165 (72%) are high confidence based on LOFTEE annotations [18]. This number of pLoF variants is consistent with the 149–182 range reported by the 1000 Genomes project [29]. The distribution of variants was more similar between genetically-inferred ancestry groups for deleterious variants than for common or less deleterious variants (Fig. 3b, Figures S8-12 and Table S9).

Determining the allele frequency of SVs is difficult due to the technical challenges of detection in population cohorts, as well as decisions around the proportion of overlap and breakpoint similarity between SVs. Recent smaller studies have estimated that a median of 98.0% of the SVs in an individual genome were common, and that SVs affected an average of around 160–180 protein-coding genes per individual genome [47, 55, 72]. In our large dataset of CNVs > 10,000 bp in 587,475 individuals, most CNVs are common: MAF > 1% CNVs represented 88.0% of the total CNV burden per individual (mean of 59.3 common CNVs per person). Across all CNVs, although most are non-coding (64.8%), on average, an individual genome has ~25 genes where a CNV is predicted to result in loss-of-function (including partial and full gene deletions, as well as gene-disruptive duplications), and ~5 genes where a duplication is predicted to result in complete copy-gain (Fig. 2b and Table S10).

### The average genome has ~63 de novo SNVs/indels, which increases with parental age at birth

**Fig. 4:**
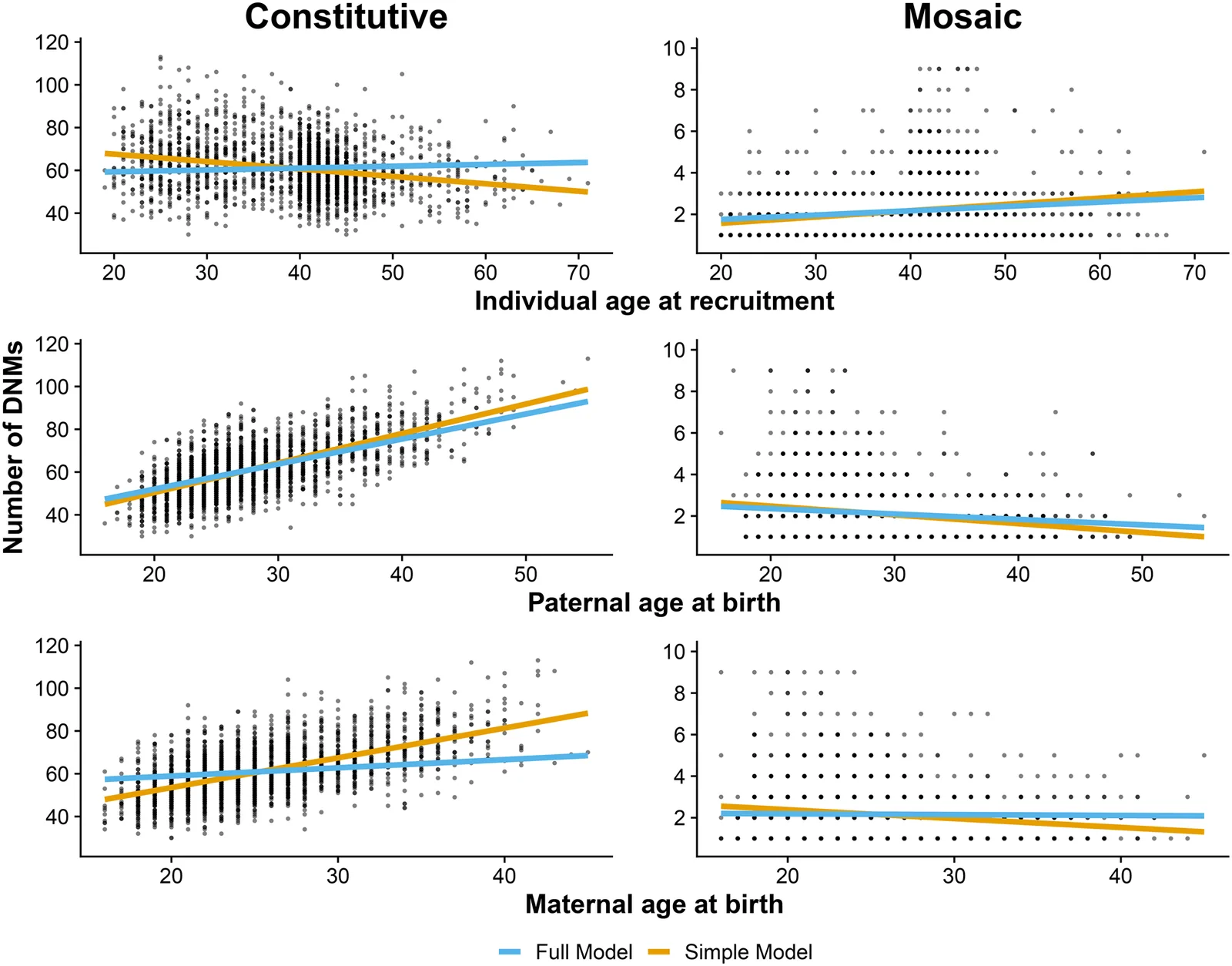
Number of *de novo* SNVs and indels per person versus age. Number of constitutive (left) and mosaic (right) *de novo* SNVs/indels in parent-offspring trios, versus age of offspring at recruitment (top), age of the father at birth (middle) and age of mother at birth (bottom). Yellow = simple linear regression (no adjustment); blue = full model (adjusted for ages of the other two members of the trio)

Using 2,334 genetically-inferred parent-offspring trios in UKB and AoU, we estimate that the average human genome has a mean of 61 constitutive *de novo* SNVs/indels (57 SNVs, 1 insertions, 3 deletions), ranging from a minimum of 30 to a maximum of 113 (Fig. 4 and Table S11). These estimates are consistent with previous studies [73–75], but lie towards the lower end of the distribution since all three individuals in a trio had to be above the cohort’s recruitment age, thus enriching for trios with young parental age at birth (mean parental age at birth of offspring was 23 years in UKB trios and 29 years in AoU trios). Constitutive *de novo* variants are mostly caused by errors during parental germ cell formation, so a lower parental age reduces the number of *de novo* variants. The number of constitutive *de novo* SNVs/indels increases by 1.17 (95%CI: [1.07, 1.27]) per year of paternal age at birth and 0.38 (95%CI: [0.27, 0.50]) per year of maternal age at birth (Fig. 4, Figures S13-14, and Table S11). Based on variants that were able to be phased across the two cohorts, ~75–80% are paternal in origin while 20–25% are maternal (Table S12 and Figure S15). The majority of *de novo* variants are non-coding, with a median of 1 (range 0–6) overlapping protein-coding exons (Figure S16).

Additionally, we further detected a median of 2 *de novo* SNVs/indels per individual that are probably mosaic (maximum allele balance 0.25) and likely result from postzygotic errors during cell division. Unlike germline constitutive variants, the number of somatic mosaic variants increases with age of the individual, by ~0.02 per year in our dataset, but not with the age of the parents (Fig. 4 and Table S12), as has been observed previously [75–78]. Somatic mosaicism is known to increase dramatically with older age due to clonal hematopoiesis [79–81]. Our estimate is likely to be an underestimate of the true levels of somatic mosaicism in these population cohorts, due to the relatively young age of individuals recruited with both parents (mean age at recruitment of offspring in trios was 39 years), and the limitations of using ~30x sequencing of mostly blood-derived and some (~5%) saliva-derived DNA.

As with all SVs, the estimated number of *de novo* SVs varies with technology, and increases with increasing resolution. Although we did not evaluate *de novo* SVs, previous studies have estimated that around 1 in 80–100 individuals have a *de novo* SV > 30,000 bp [82], whilst 1 in 6–7 individuals has a *de novo* SVs > 20 bp, which (excluding chromosomal aneuploidies) were also mostly paternally-derived [83, 84]. A recent study that used multiple orthogonal technologies in a four-generation pedigree estimated an additional ~65 *de novo* SVs per person originating from tandem repeats, taking the total *de novo* variant burden to 95–178 per individual [78].

### The average person has ~1–3 rare ClinVar pathogenic/likely pathogenic variants linked with monogenic diseases

**Fig. 5:**
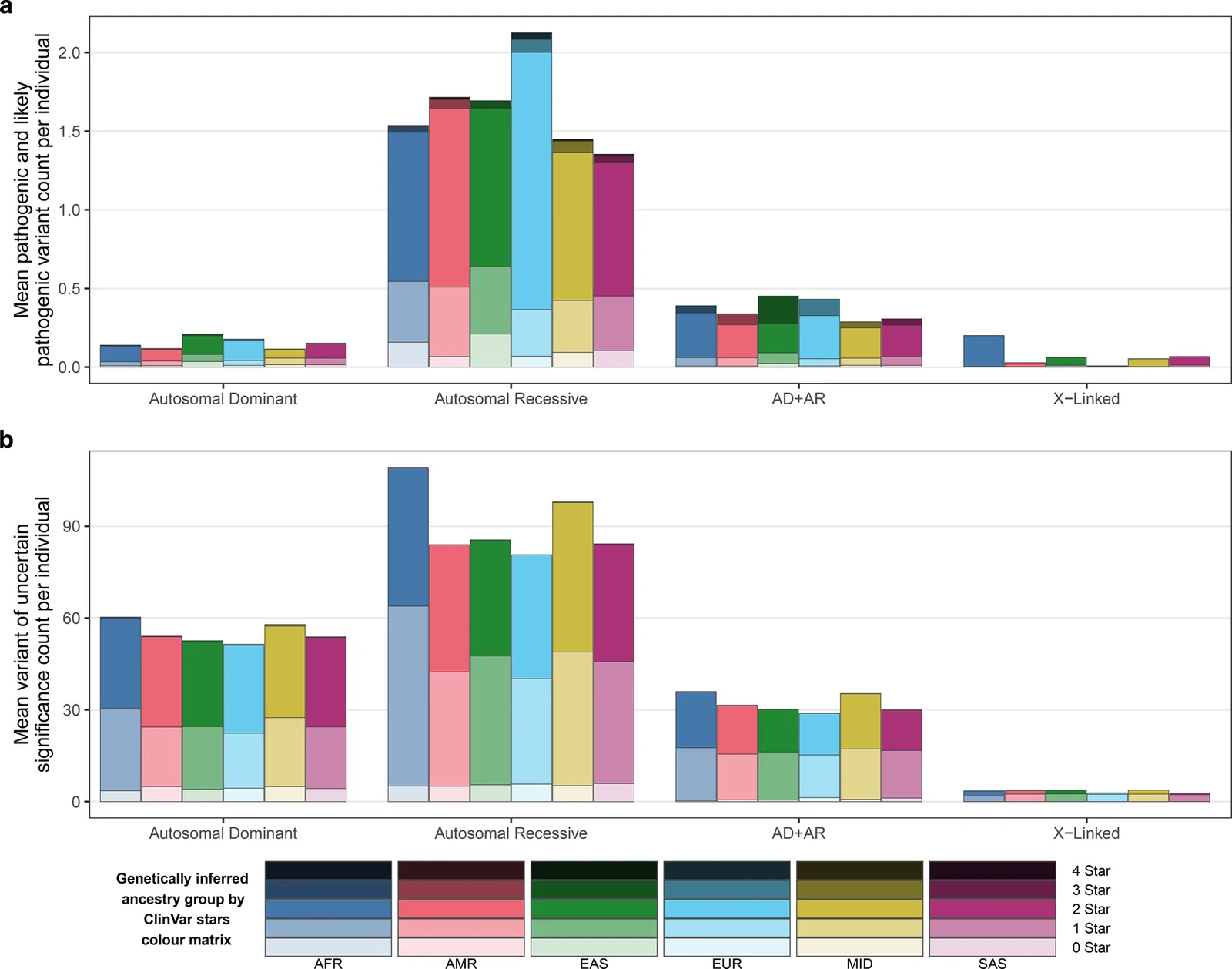
Distribution of ClinVar variants in GenCC-curated monogenic disease-associated genes, split by modes of inheritance. **a**) number of ClinVar pathogenic/likely pathogenic variants per genome; **b**) number of ClinVar variants of uncertain significance (VUS) per genome. GenCC-curated disease genes were included if the genes were supported by definitive, strong or moderate levels of evidence without the associations being refuted or disputed (Table S4). ClinVar star-rating relates to the level of review supporting the variant classification (4 = practice guideline; 3 = reviewed by expert panel; 2 = criteria provided, multiple submitters, no conflicts; 1 = criteria provided, single submitter; 0 = no assertion criteria). AD = autosomal dominant; AR = autosomal recessive; AD+AR = both AD and AR conditions

At the date of analysis (8 December 2025), the ClinVar database contained ~4 M variants [37]. Of these, ~300,000 were rated pathogenic or likely pathogenic (P/LP) in the germline, of which 8–10 are present in the average human genome, consistent with previous recent reports [85] (Figure S17). Given that the majority of protein-coding genes remain without a known role in human development or disease, we limited our analysis to monogenic disease-associated genes from GenCC (*n* = 4,262 genes) [38], consequences annotated in MANE Select and MANE Plus Clinical transcripts, and curated variants with MAF > 1% to exclude risk alleles. This analysis reduces the number of P/LP variants per person to ~1–4 (mean 1.9–2.6), of which the majority are in genes linked only with autosomal recessive diseases, consistent with previous estimates of carrier status [86] (Fig. 5a, Figure S18 and Table S13). Interestingly, variants that were rated 2-star by ClinVar (multiple submitters, no conflicts) were most frequent in the average genome. This is influenced by high-frequency variants in well-established conditions with higher allele frequencies within specific populations [87, 88], such as haemochromatosis (*HFE* p.Cys282Tyr, MAF of 7–8% in UKB-EUR), G6PD-deficiency (*G6PD* p.Val68Met, MAF of 12% in UKB-AFR), and the haemoglobinopathies (*HBB* p.Glu7Val and p.Glu7Lys, MAF of 8 and 2% in UKB-AFR, respectively); truncating variants in filaggrin (*FLG*), known to be associated with eczema and ichthyosis with incomplete penetrance [89], were also present in around 1 in 15 EUR-like individuals. Limiting to variants with MAF < 1% does not substantially alter the average number of P/LP variants per person (~1–3, Figure S19 and Table S14).

Many monogenic diseases are caused by very rare damaging variants not present in ClinVar or other databases of disease-associated variation, therefore rare pLoF variants are routinely included in diagnostic analyses [90, 91]. Considering just MANE Select and MANE Plus Clinical transcripts in the same GenCC-curated gene list, on average individuals carry 19–29 high-confidence pLoF variants (2–5 with MAF < 1%) in monogenic disease-associated genes (Figures S20–23 and Tables S15-16). This high number suggests that many are not truly loss-of-function and require further curation as they may be false positives [92, 93] or be in genes associated with a gain-of-function or dominant negative pathomechanism [94]. Nonetheless, limiting to only rare variants (MAF < 1%) in GenCC genes associated exclusively with autosomal dominant diseases, on average around 1 in 5–11 individuals have at least one rare ClinVar P/LP variant (1 in 8.6 on average, or 1 in 12.8 on average limiting to ≥ 2-star) and 1 in ~2–3 individuals have a rare high-confidence pLoF variant. Given the healthy-volunteer bias of population biobanks [69, 95], even true loss-of-function variants may be non-penetrant due to genetic or environmental modifiers [96, 97]. Individuals may also be heterozygous carriers of ~2–6 autosomal recessive conditions (~0–3 rare ClinVar P/LP variants and ~1–4 rare HC pLoF variants), and 1 in ~13–37 females are carriers of an X-linked condition (1 in ~18–100 carry rare ClinVar P/LP variants and 1 in ~42–56 carry rare HC pLoF variants).

Variants of uncertain significance (VUS) numbered ~2 million in the ClinVar database (~7-times more than LP/P variants), but occur ~30–90-times more frequently than P/LP variants in the average genome, with ~180 per person (~50 per person with MAF < 1%) in GenCC-curated genes (Fig. 5b, Figures S19, S24-25 and Tables S13-14). There is a notable difference in the trend between P/LP variants and VUS by genetically-inferred ancestry group, whereby EUR-like individuals have more P/LP variants – and thus are more likely to receive a diagnosis – whilst non-EUR-like individuals have notably more VUS. This bias has been noted before [90, 98–100], and can be explained by a number of contributing factors: first, differing allele frequencies across different populations (i.e. a rare variant in one population may be common in another, and thus present in more individual genomes); second, lack of diversity in reference datasets (i.e. variants that are common in well-represented populations are easier to classify as benign or likely benign) [101]; and third, better characterisation of P/LP variants in EUR-like individuals (i.e. due to differences in accessing genomic testing between countries) [102]. It should be noted that this analysis is limited to VUS catalogued in ClinVar at the date of analysis, not all possible VUS in a genome, which will affect both the number of variants and the differences between groups.

### The average person has a high polygenic score for at least one common complex disease

**Fig. 6:**
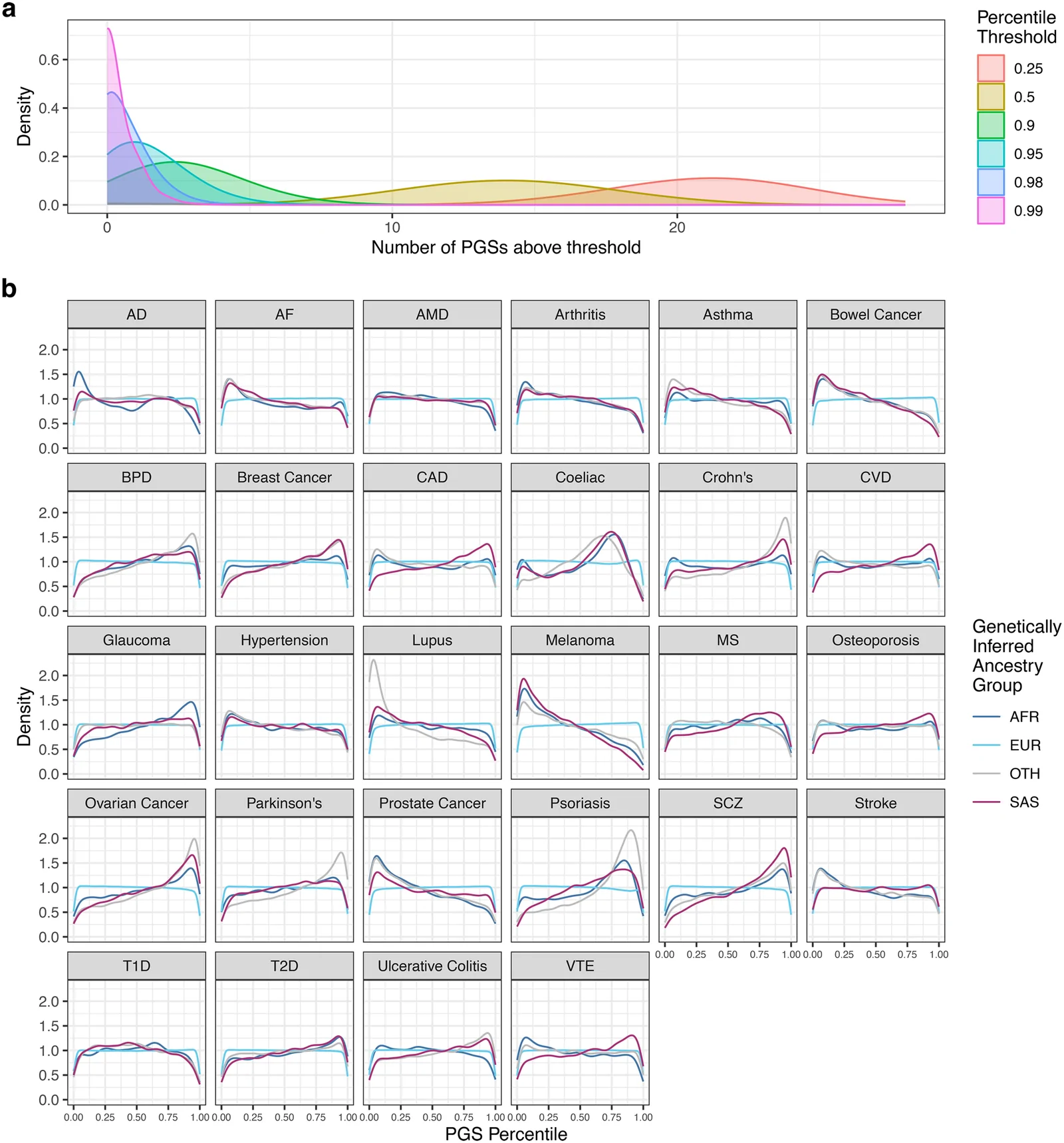
Distribution of polygenic risk score percentiles. **a**) distribution of the number of polygenic risk scores (PGSs) exceeding 25th, 50th, 90th, 95th, 98th, and 99th centile in UKB participants. **b**) distribution of PGS percentile, stratified by PGS and genetically-inferred ancestry group; PGS scores that are consistent across all groups will have flat probability densities, whilst those that perform differently at the extremes of risk and are non-normally distributed will be skewed; x-axis represents the PGS percentile, y-axis represents the density for each PGS within each population. AD = Alzheimer’s Disease; AF = atrial Fibrillation; AMD = age-related macular Degeneration; BPD = BiPolar Disorder; CAD = coronary artery Disease; CVD = cardiovascular Disease; MS = multiple Sclerosis; SCZ = Schizophrenia; T1D = type 1 Diabetes; T2D = type 2 Diabetes; UC = ulcerative Colitis; VTE = venous thromboembolic disease

We used polygenic scores (PGS) for 28 common complex diseases that have been pre-calculated in UKB (Table S3) [103] to estimate the proportion of common multifactorial diseases for which an individual might be at high polygenic risk, irrespective of disease heritability. The average person is in the top 10% of the PGS for three conditions and in the top 5% for a mean of 1.4 conditions (Fig. 6a). Among the 450,209 UKB-EUR participants, 102,831 individuals (i.e. ~1 in 5 people) have a score in the top 1% for at least one PGS. Among the 35,844 non-EUR participants in UKB, 10,806 had a score in the top 1% (~1 in 3 people). This higher number of PGSs in the top 1% in non UKB-EUR participants is primarily driven by differences in distributions of PGSs in these participants (Fig. 6b), i.e. it is caused mainly by a small subset of PGSs where individuals fall disproportionately within the top 1% compared to UKB-EUR, due to differences in the frequency of particular contributing SNPs across ancestries [29]. This difference is likely to be artefactual with respect to disease risk, and reflects poor performance of PGS in non-EUR populations [104] – in some cases vastly exceeding differences in disease prevalence [105] – which underscores the fact that PGS derived from one population should not be carelessly applied to another without thorough evaluation.

Given the continuous nature of PGS [106], the distributions in UKB-EUR participants follow the expected proportion of individuals with at least one PGS in the top 1%, under the assumption that the probability of each PGS being in the top 1% is 0.01 for each individual-PGS pair (assuming each PGS is independent, see Figure S26). Following these assumptions, if 100 PGS were calculated, then ~99% of individuals would be in the top 5% for at least one disease, and ~63% would be in the top 1%. Individuals could therefore expect to have five PGS in the top 5% and 1 in the top 1%.

## Discussion

The last decade of human genetics has resulted in a new appreciation of the extent of genetic variation present in every individual human genome sequence. Of the ~4.3–5.3 million variants in an individual genome, the vast majority are common, non-coding, and unlikely to have a measurable phenotypic effect. Every individual genome also has a small number of rare loss-of-function variants and novel *de novo* coding variants, as well as known pathogenic variants in disease-associated genes [107], most of which do not cause disease in that individual. This raises questions about the clinical value of reporting such findings outside of a diagnostic context, due to high carrier frequencies and incomplete penetrance. Recognition of this background variation is important for genomic scientists interpreting sequence data and for clinicians counselling patients about novel findings and variants of uncertain significance [108], and for policy-makers deciding which variants should be routinely reported in individuals undergoing sequencing [109]. A cautious approach should also be taken to interpreting large structural variants in older individuals due to age-related somatic clonal expansion, which may predominate over germline variants in some regions of the genome [79, 110]. Every genome also has several orders of magnitude more VUS than pathogenic variants, and this uncertainty is unequally distributed between ancestry groups, potentially exacerbating health disparities [102]. Moreover, most people have a high polygenic score for at least one disease, which – if multiple PGSs were implemented simultaneously in a screening context – has significant potential for overdiagnosis, particularly for conditions with low heritability [111], and overburdening healthcare systems.

The huge increase in number of individuals sequenced over the last 20 years has resulted in a concomitant increase in the number of rare variants catalogued and a corresponding decrease in the number of rare and novel variants in an individual genome. This trend will likely continue as more individuals are sequenced and their data added to shared federated repositories [112, 113]. To the best of our knowledge, this is the first time that such a large-scale and comprehensive survey of the genome-wide distribution of variation within an individual (as opposed to a cohort) has been assembled into a single publication, including rare, common, small, large, coding, non-coding, *de novo* and disease-relevant variants. We found highly consistent results across ~900,000 individuals in two independent biobanks, thanks in part to the similar technologies used to generate WGS and the overlapping timeframes during which the datasets were analysed. Where possible, we have included cross-ancestry comparisons and highlighted important differences, adding strength and wider applicability to our estimates. Nonetheless, whilst our study represents the largest analysis to date that attempts to summarise the variation present in the “average” individual genome, there are several important limitations.

Firstly, the two cohorts we used – UKB and AoU – are limited in geographical scope (to the UK and USA, respectively), and lack individuals from the majority of geographical regions and genetic ancestries globally [114]. Whilst we categorise individuals in our study as being genetically similar to previously defined genetically-inferred ancestral groupings (Supplementary Note S1), we acknowledge that these groupings are themselves reductive and a limited measure of global genetic diversity [115, 116]. Although humans are more similar than we are different, and the vast majority of common variation has likely already been found, variation present only in indigenous and geographically-isolated populations will be entirely absent from existing databases and cohorts. The ~25% increase in the amount of variation observed in 1KG-AFR-like individuals versus 1KG-EUR-like is a stark reminder of the perils of ignoring genetic diversity. Furthermore, even within this category, 1KG-AFR-like individuals from UKB are likely to be much less admixed than those in AoU (Figure S2), underlining the need for data from representative populations to facilitate less crude ancestry-based groupings. The underrepresentation of particular genetic ancestries has been shown to result in poorer diagnostic yields in monogenic disease [90, 99, 100] and lower predictive performance of PGS in common diseases [101, 104, 117], which further underscores the urgent need to increase diversity across genetic datasets [118] and social contexts [119] to enable equitable precision medicine.

Secondly, our analysis was limited to WGS data generated primarily (~95%) from blood-extracted DNA using short-read next-generation DNA sequencing technology. Whilst the accuracy for detecting SNVs and small indels is extremely high using this technology, accurately detecting SVs remains challenging and some types of variants (e.g. inversions, mobile-element insertions, tandem repeats) are almost impossible to detect reliably at population scale [120]. Moreover, sequencing blood-derived DNA at ~30x depth means that somatic variation present in other tissues (or at a very low-level in blood) will be missed. Ideally, to fully sample the breadth of human genetic variation, these datasets would need to be coupled with long-read sequencing [121] and sequencing of multiple tissues [122]. Furthermore, aligning sequence reads to complete telomere-to-telomere (“T2T”) pangenome reference sequences [123, 124] would improve detection of all types of variation across ancestries [125, 126]. Notably, data from a recent study using five complementary short-read and long-read sequencing technologies aligned to T2T across a four-generation pedigree was able to detect more than double the number of *de novo* variants compared with other studies, primarily as a result of improved detection of tandem repeats [78].

The next decade will see a dramatic increase in the number of individual genome sequences generated. This expansion should be accompanied by greater geographical and ancestral diversity, led by numerous major initiatives across the globe [114, 127–130]. However the ability to utilise these more diverse samples to reduce inequity is contingent on global data sharing [131]. Over this time period, the use of population-specific pangenomes will likely become the norm and will improve genome assembly and genotyping particularly of structural variants [132, 133]. Genome sequencing data will be increasingly linked to phenotype information, including electronic health records, whilst functional genomics [134] and advances in AI [135, 136] will continue to improve prediction of variant consequences, and should reduce the number of VUS [137]. Whilst these developments will improve our ability to interpret genetic variants, the ubiquity of disease-associated variants in the “healthy” genome underlines the need for caution as we move towards an era of genomic-driven preventative health strategies and precision medicine. Ultimately, a balance must be found between the potential benefits of reporting clinically actionable findings to individuals versus the potential harms of mis-diagnosis and overdiagnosis [138], as well as the costs of returning genomic results to individuals [139].

## Conclusions

Although much genomics research to date has – rightly – focused on patients with disease, it is of paramount importance to understand the context in which disease-associated variation is identified. Indeed, the availability of aggregate variant data in gnomAD from cohorts spanning hundreds of thousands of individuals has already been transformative across genomic medicine and research [140, 141]. Using individual-level WGS data from adult population biobanks provides a rich source of variation observed in the “healthy” human genome. We have summarised the state of knowledge of individual genomic variation in 2026, by analysing genomes from nearly a million individuals from two population cohorts in the UK and USA, covering six genetically-inferred ancestry-similarity groups, with data from short-read DNA sequencing technology that is currently standard throughout the world. Our findings highlight the ubiquity of human genetic variation and should inform anyone – clinicians, scientists, policy-makers, patients, and the general public – trying to navigate the translation of human genomic knowledge into precision medicine.

## Electronic supplementary material

Below is the link to the electronic supplementary material.

Supplementary Material 1:
Additional File 1

Supplementary Material 2:
Additional File 2

## Data Availability

The datasets supporting the conclusions of this article are available in the UK Biobank and All of Us research analysis platforms.
