# Supplementary material for: The average individual genome: a survey of nearly 1 million whole-genome sequences
Source: Genome Med. 2026 Oct 22;18:160. doi: 10.1186/s13073-026-01792-x (PMC13689340; doi:10.1186/s13073-026-01792-x)
Supplement: Supplementary file 1 [file 13073_2026_1792_MOESM1_ESM.pdf]

Supplementary Note and Figures

Table of contents

Supplementary Note S1........2
Supplementary Note S2........2
Figure S1: Distribution of age at biosample collection in AoU (top) and recruitment in UKB (bottom) split by genetically inferred ancestry group and sex........3
Figure S2: Distributions of the number of reference calls and missing calls per individual in AoU (top) and UKB (bottom)........4
Figure S3: GIAB overlap fraction versus MAF for jointly-called copy number variants of length >1 Mb in AoU........5
Figure S4: Population-level summaries of CNVs........6
Figure S5: Distributions of ROH in AoU........7
Figure S6: Distributions of ROH in UKB........8
Figure S7: Density of ROH across the genome........9
Figure S8: Number of SNVs and indels per individual in AoU (top) and UKB (bottom), stratified by allele frequency........10
Figure S9: Number of SNVs and indels per individual in AoU stratified by genome-wide worst consequence........11
Figure S10: Number of SNVs and indels per individual in UKB stratified by genome-wide worst consequence........12
Figure S11: Number of SNVs and indels per individual in AoU stratified by worst consequence within MANE Select transcripts........13
Figure S12: Number of SNVs and indels per individual in UKB stratified by worst consequence within MANE Select transcripts........14
Figure S13: Number of constitutive and mosaic de novo variants in AoU........15
Figure S14: Number of constitutive and mosaic de novo variants in UKB........16
Figure S15: Number of paternally and maternally phased constitutive de novo variants........17
Figure S16: The number of constitutive de novo SNV/Indels within genomic regions........18
Figure S17: Initial Curation of ClinVar Variants........19
Figure S18: Percentage of individuals carrying pathogenic or likely pathogenic ClinVar variants........20
Figure S19: Mean number of MAF < 1% ClinVar variants in GenCC-curated monogenic disease-associated genes carried by individuals in AoU........21
Figure S20: Mean number of high-confidence loss-of-function variants in GenCC-curated monogenic disease associated-genes carried by individuals........22
Figure S21: Mean number of MAF < 1% high-confidence loss-of-function variants in GenCC-curated monogenic disease associated-genes carried by individuals in AoU........23
Figure S22: Percentage of individuals carrying high-confidence loss-of-function variants in GenCC genes........24
Figure S23: Percentage of individuals in AoU carrying MAF < 1% high-confidence loss-of-function variants in GenCC genes........25
Figure S24: Percentage of individuals carrying ClinVar variants of uncertain significance........26
Figure S25: Percentage of individuals in AoU carrying MAF < 1% ClinVar variants of uncertain significance........27
Figure S26: Correlation between PGSs in UKB........28

Supplementary Note S1

In many places within this supplementary information and the main paper we stratify results by genetically-inferred ancestry group. These groups are defined by genetic similarity with 1000 Genomes Project and/or Human Genome Diversity Project individuals (see main methods), which are themselves grouped into six broad population groups matched in our data (AFR = African, AMR = Admixed American, EAS = East Asian, EUR = European, MID = Middle-Eastern, SAS = South Asian). As such, when we stratify individuals into groups in our results, we are not grouping by ancestry (which may be understood on an individual level to be “a person’s origin or descent, lineage, “roots,” or heritage, including kinship” [1]), but by varying amounts of genetic similarity with six broad and reductive groups. We believe that this stratification is valuable because it can highlight differences in our understanding of genetic variation originating in different populations. A recent report [1] provides detailed examination of the history of and suggested best practices for using population descriptors, which we used as guidance in writing this paper.

1. National Academies of Sciences, Engineering, and Medicine. *Using Population Descriptors in Genetics and Genomics Research: A New Framework for an Evolving Field* (National Academies Press, 2023).

Supplementary Note S2

Due to data access restrictions, total variants per individual could not be exported directly from UKB, so the overall per-person counts in main Figure 1 were reconstructed from the per-category counts (SNV/indel × genotype/region). Because the overall distribution depends on the covariance between each category (autosome homozygous, chrX haploid, etc..), we estimated this covariance in AoU as a per-sex correlation matrix. We then sampled per-individual category counts jointly from the combined binned data with a Gaussian copula (using the AoU variant-category correlation matrix and each category's exported marginal distribution) and summed them to obtain overall per-individual counts. This method reproduces both the marginal counts and their covariance. Per-category panels were still plotted directly from the observed data, except the male-PAR panel where the heterozygous and homozygous categories are combined by the same copula sampling.

Supplementary Figures

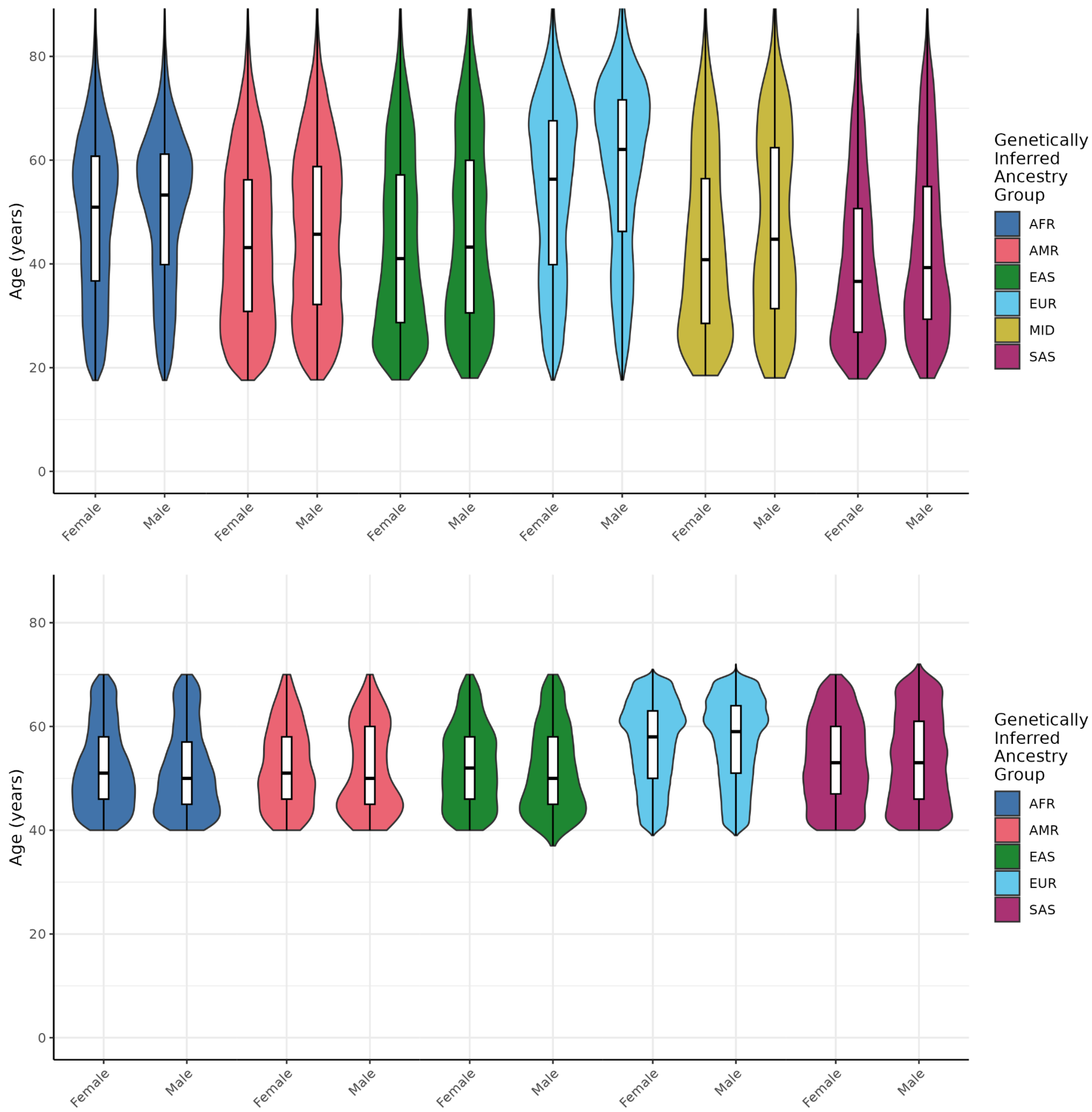

Figure S1: Distribution of age at biosample collection in AoU (top) and recruitment in UKB (bottom) split by genetically inferred ancestry group and sex.

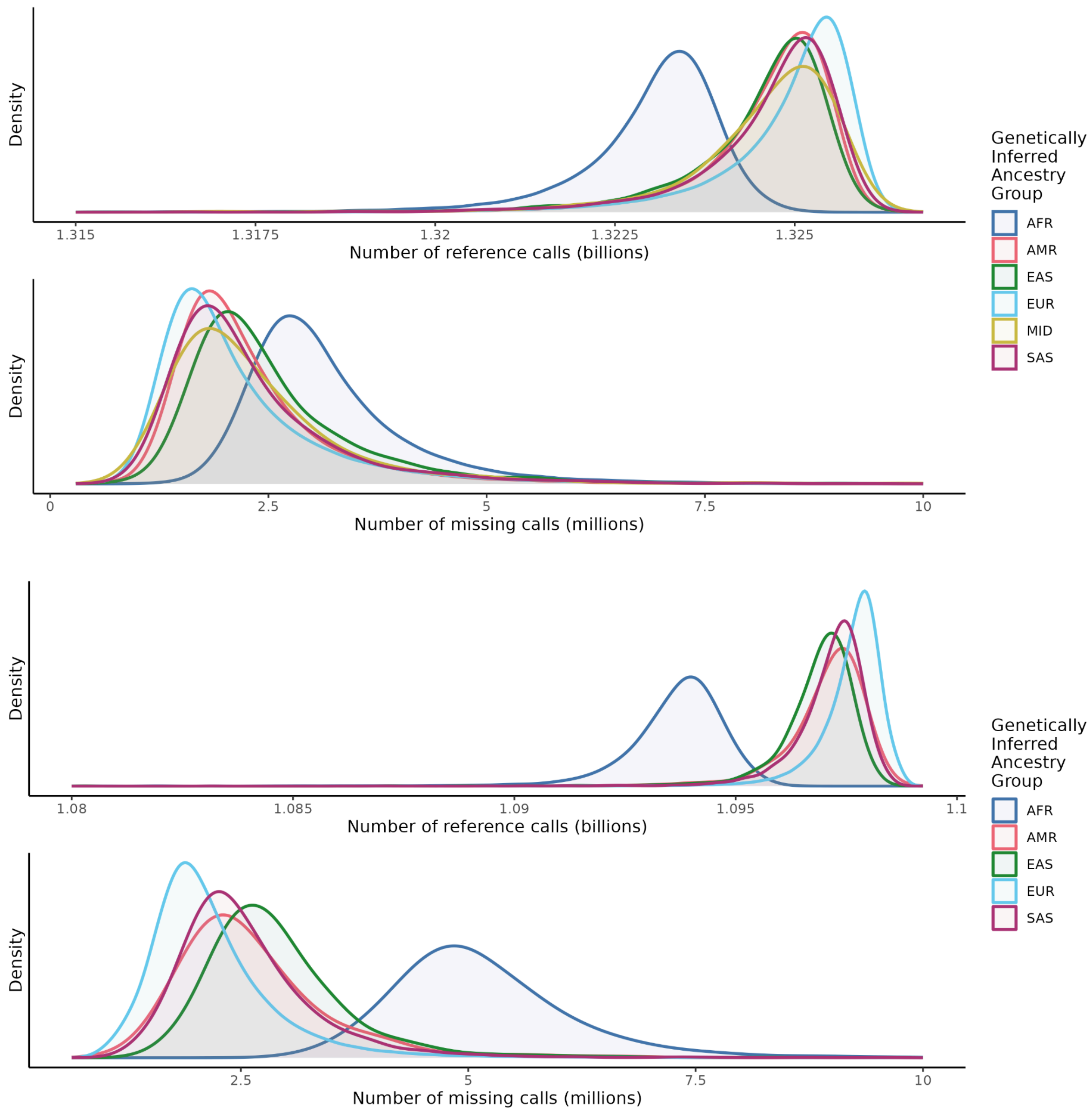

Figure S2: Distributions of the number of reference calls and missing calls per individual in AoU (top) and UKB (bottom).

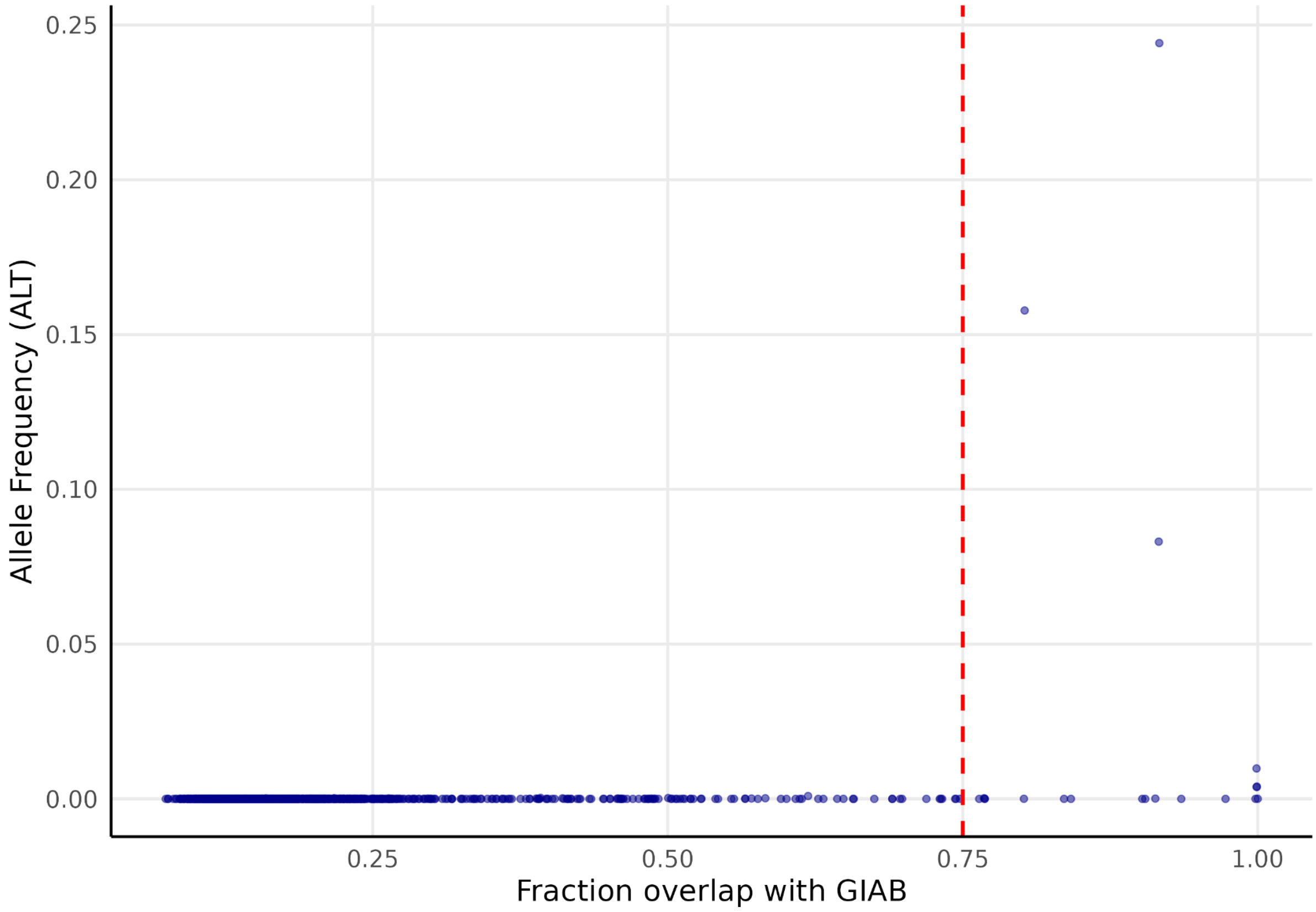

Figure S3: GIAB overlap fraction versus MAF for jointly-called copy number variants of length >1 Mb in AoU.

Red line denotes 0.75, which we chose to rule out the possibility of a common megabase-deletion

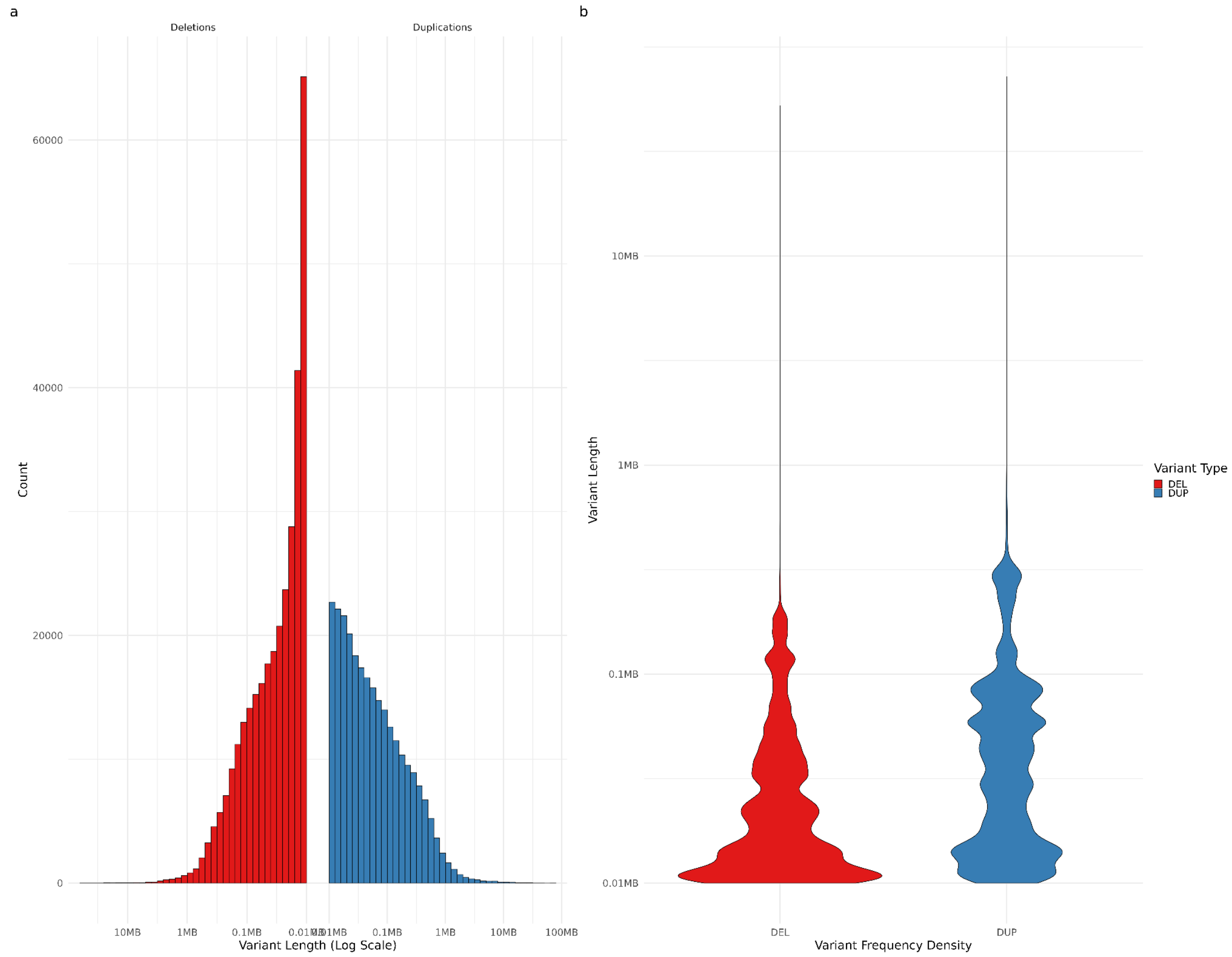

Figure S4: Population-level summaries of CNVs.

a) Distribution of CNV sizes after quality control (VCF QUAL filter >5th centile per biobank and per CNV type, and GIAB overlap < 75%); b) violin-plot distribution of CNV frequency, calculated across all individuals, versus CNV length in basepairs and stratified by type. Red = DEL (deletion); blue = DUP (duplication).

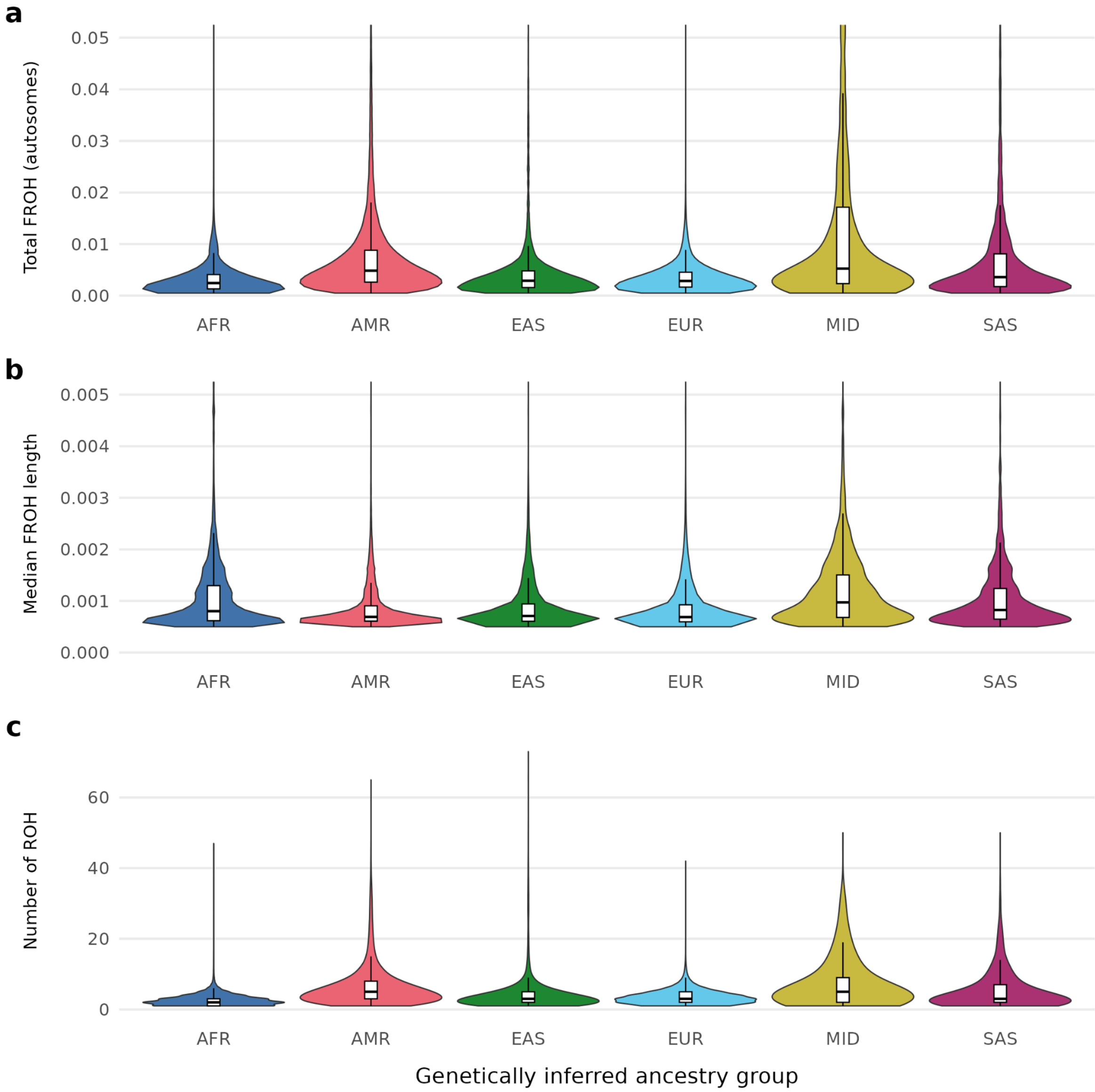

Figure S5: Distributions of ROH in AoU.

Violin plots showing a) total proportion of the genome made up of runs of homozygosity b) median length of runs of homozygosity as a proportion of the genome, and c) number of runs of homozygosity per person on autosomes. AFR = African, AMR = Admixed American, EAS = East Asian, EUR = European, MID = Middle-Eastern, SAS = South Asian.

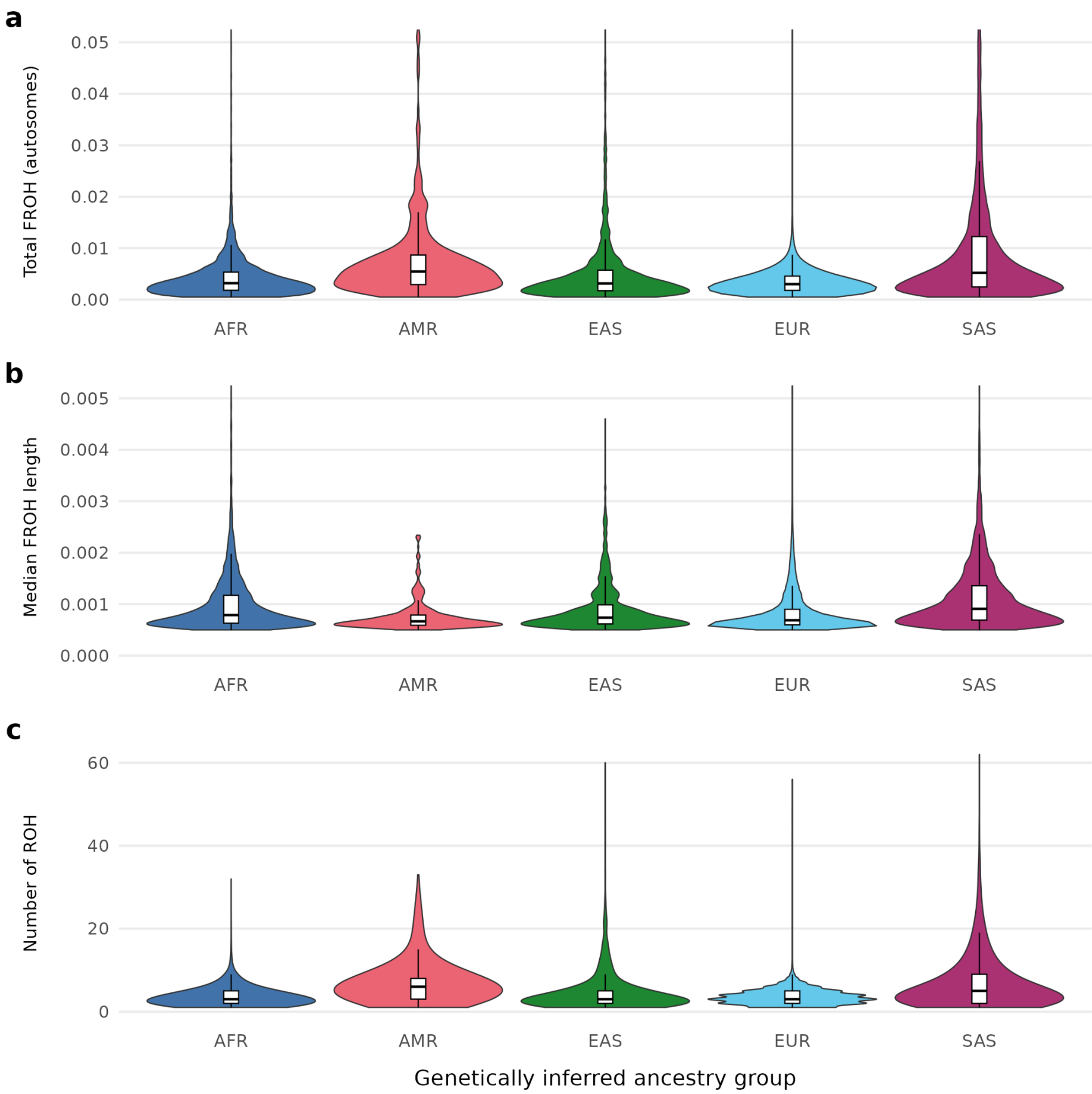

Figure S6: Distributions of ROH in UKB.

Violin plots showing a) total proportion of the genome made up of runs of homozygosity b) median length of runs of homozygosity as a proportion of the genome, and c) number of runs of homozygosity per person on autosomes. AFR = African, AMR = Admixed American, EAS = East Asian, EUR = European, SAS = South Asian.

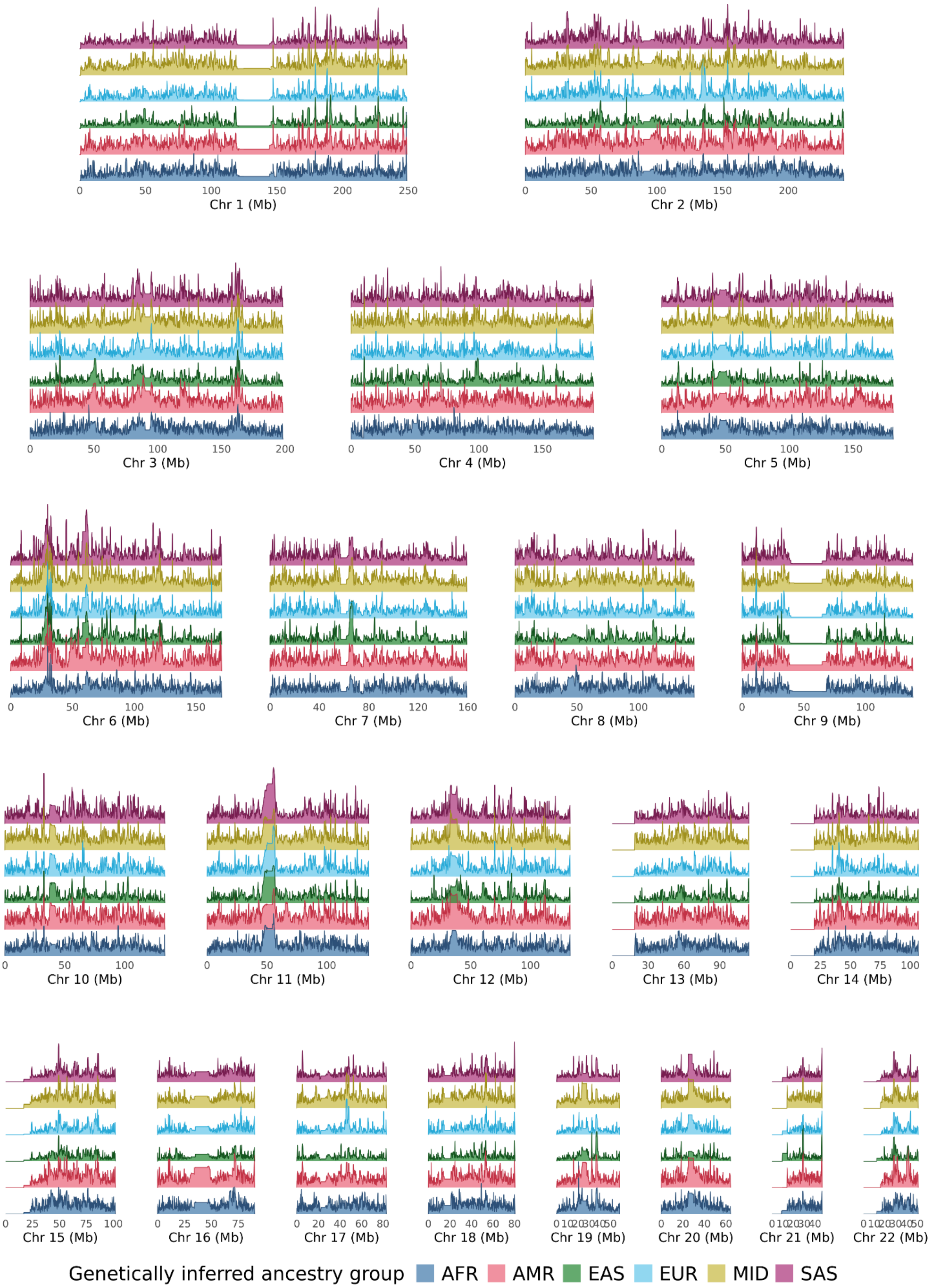

Figure S7: Density of ROH across the genome.

Proportion of genotype calls across the genome which fall into a ROH, stratified by ancestry groups**.** AFR = African, AMR = Admixed American, EAS = East Asian, EUR = European, MID = Middle-Eastern,SAS = South Asian.

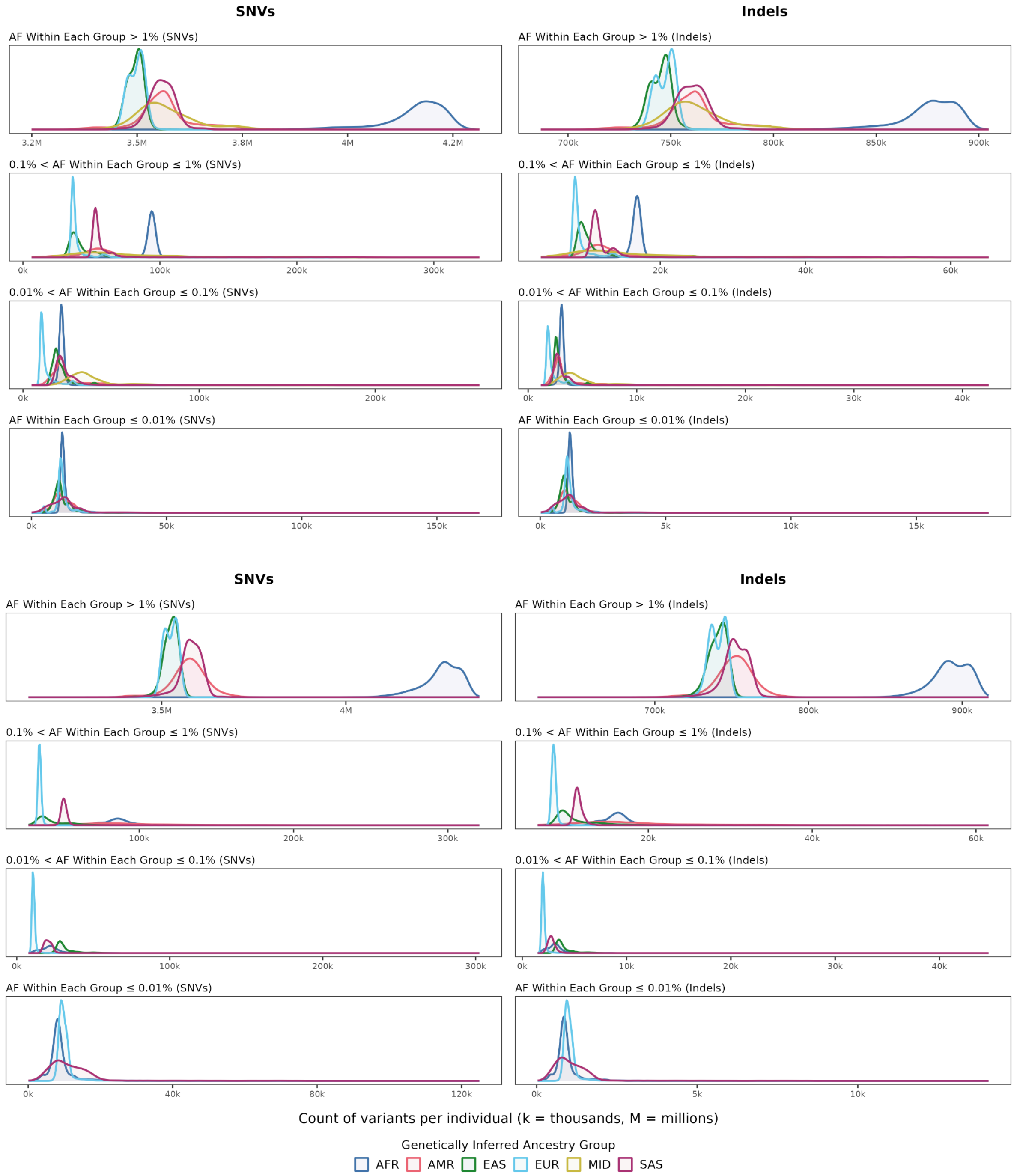

Figure S8: Number of SNVs and indels per individual in AoU (top) and UKB (bottom), stratified by allele frequency.

AFR = African, AMR = Admixed American, EAS = East Asian, EUR = European, MID = Middle-Eastern, SAS = South Asian.

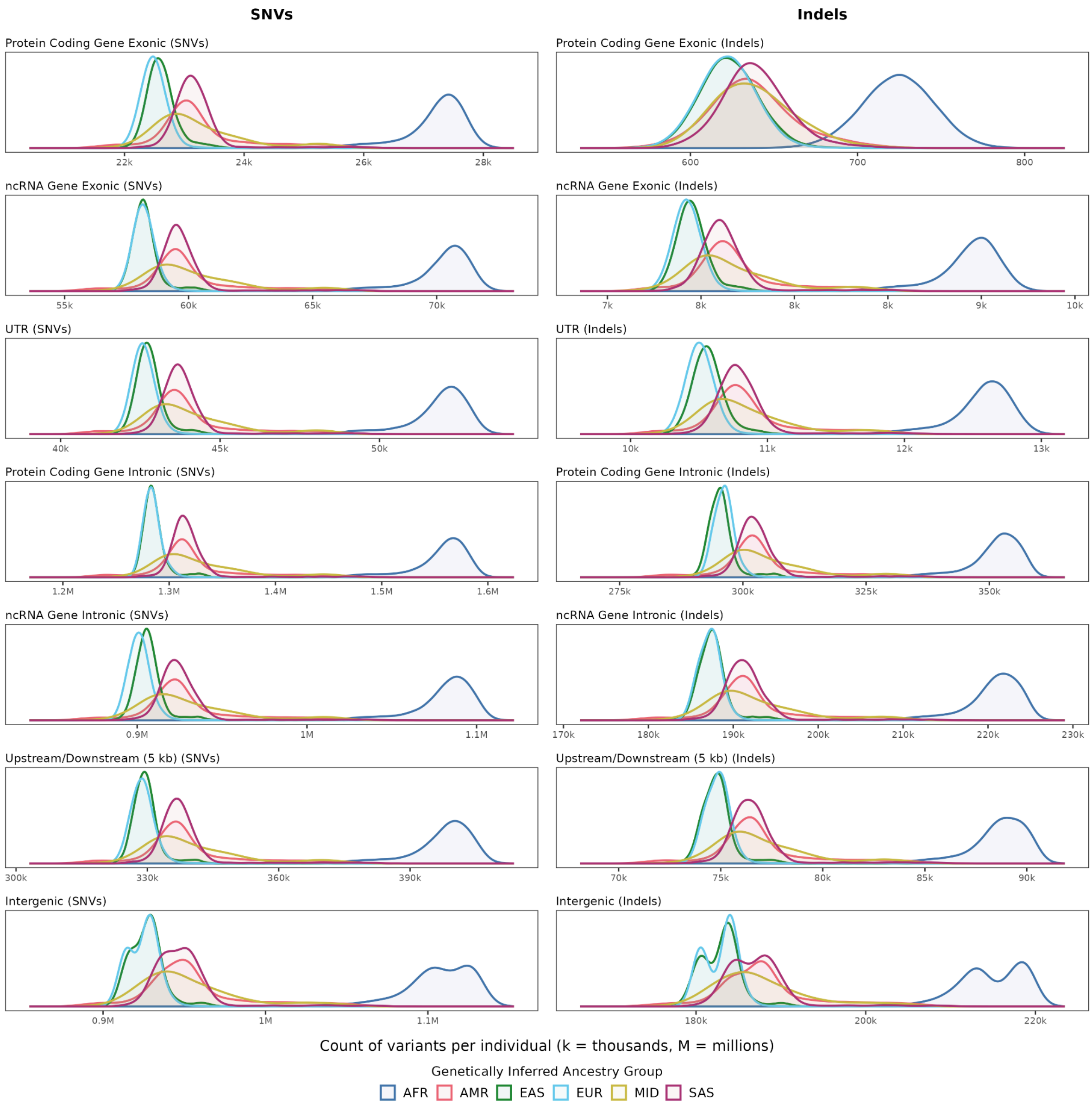

Figure S9: Number of SNVs and indels per individual in AoU stratified by genome-wide worst consequence.

AFR = African, AMR = Admixed American, EAS = East Asian, EUR = European, MID = Middle-Eastern, SAS = South Asian.

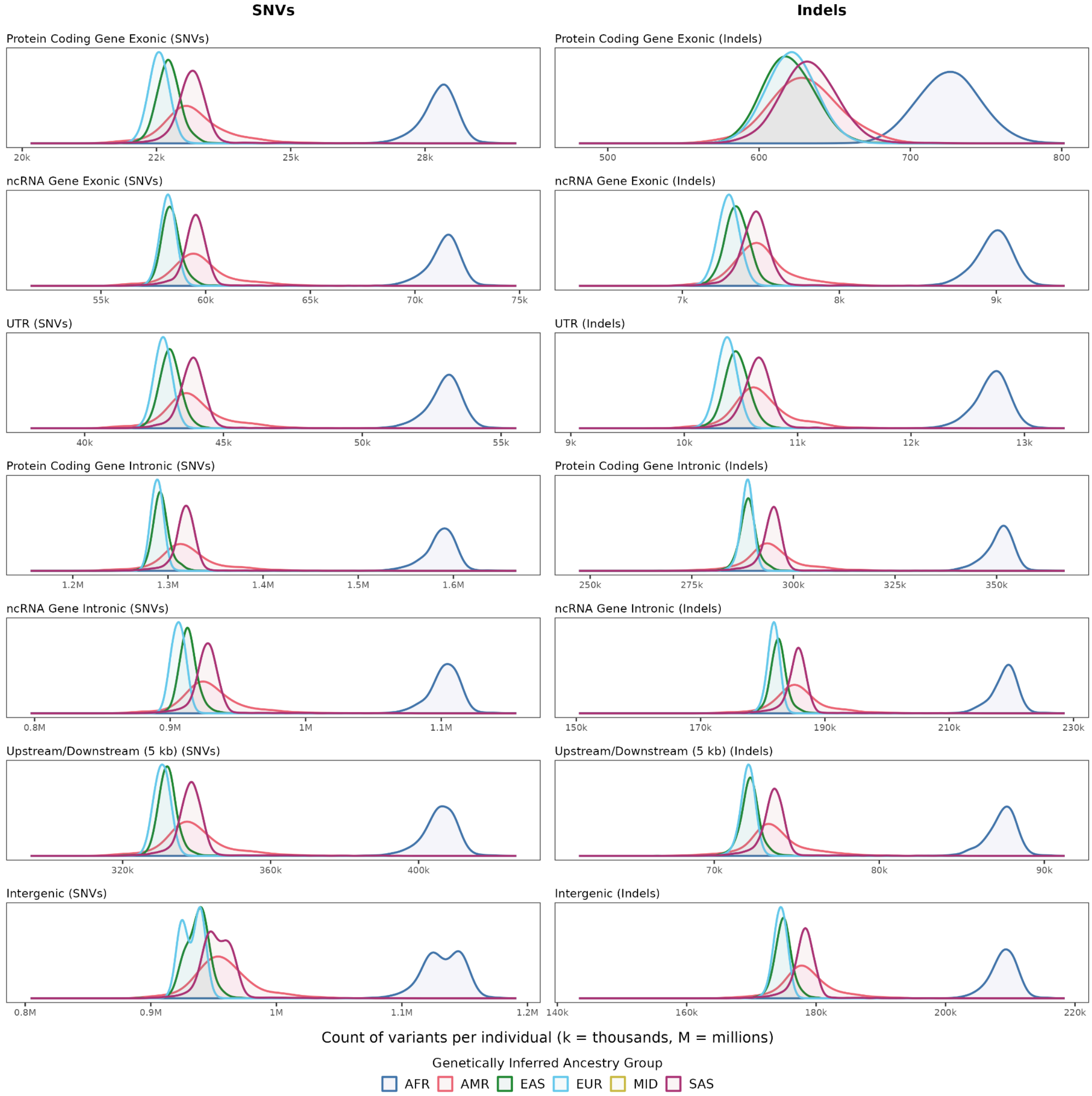

Figure S10: Number of SNVs and indels per individual in UKB stratified by genome-wide worst consequence.

AFR = African, AMR = Admixed American, EAS = East Asian, EUR = European, MID = Middle-Eastern, SAS = South Asian.

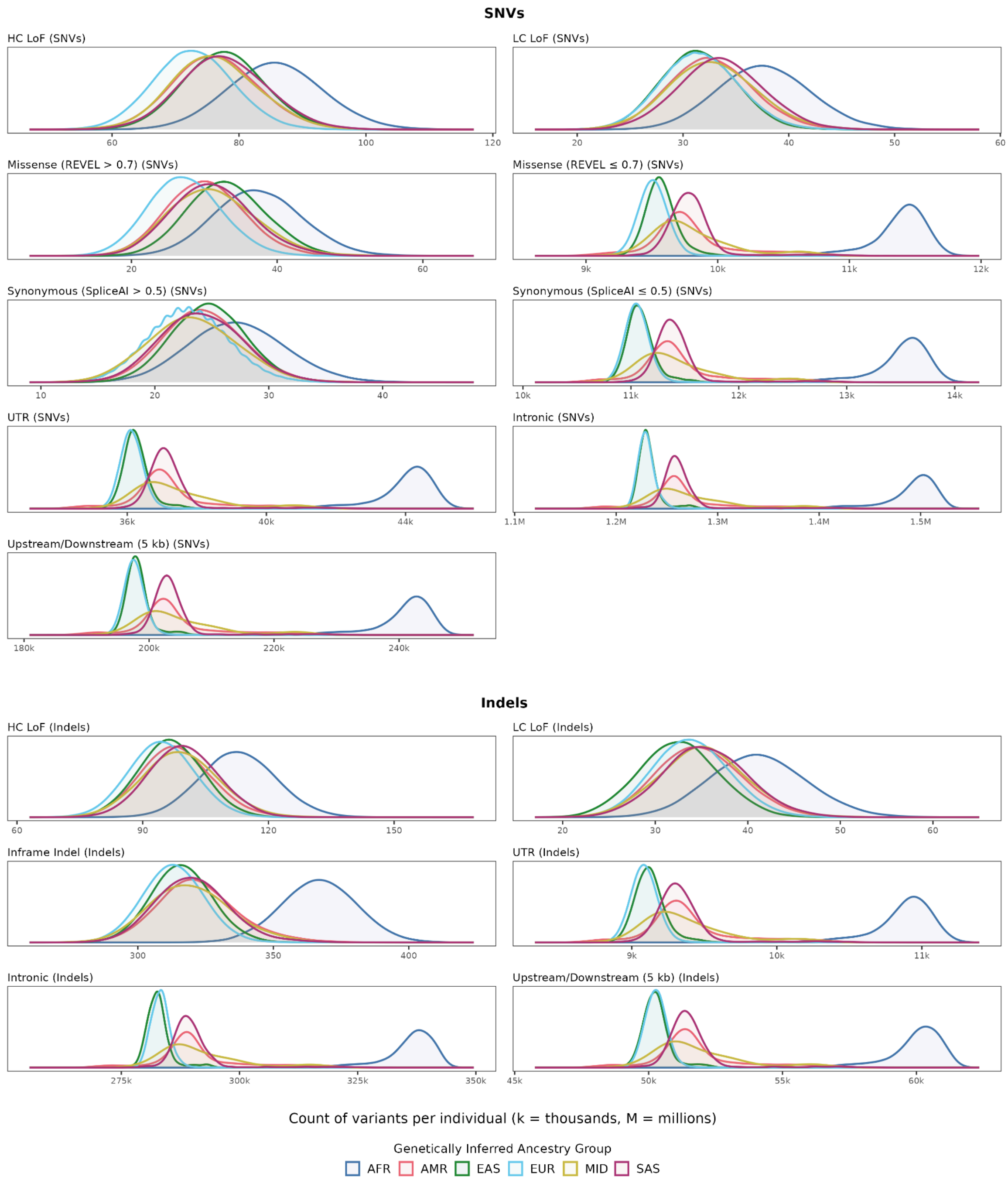

Figure S11: Number of SNVs and indels per individual in AoU stratified by worst consequence within MANE Select transcripts.

AFR = African, AMR = Admixed American, EAS = East Asian, EUR = European, MID = Middle-Eastern, SAS = South Asian.

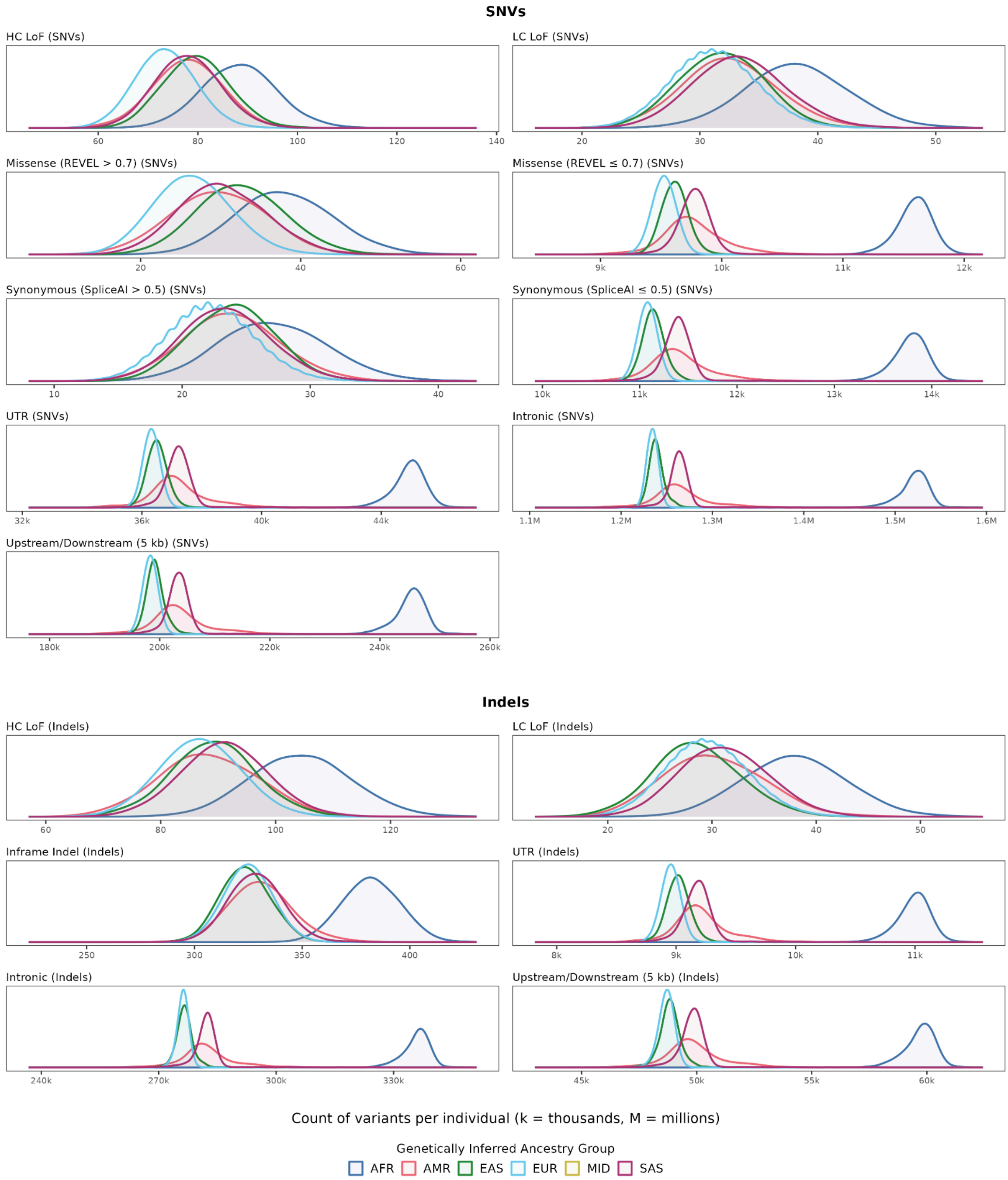

Figure S12: Number of SNVs and indels per individual in UKB stratified by worst consequence within MANE Select transcripts.

AFR = African, AMR = Admixed American, EAS = East Asian, EUR = European, MID = Middle-Eastern, SAS = South Asian.

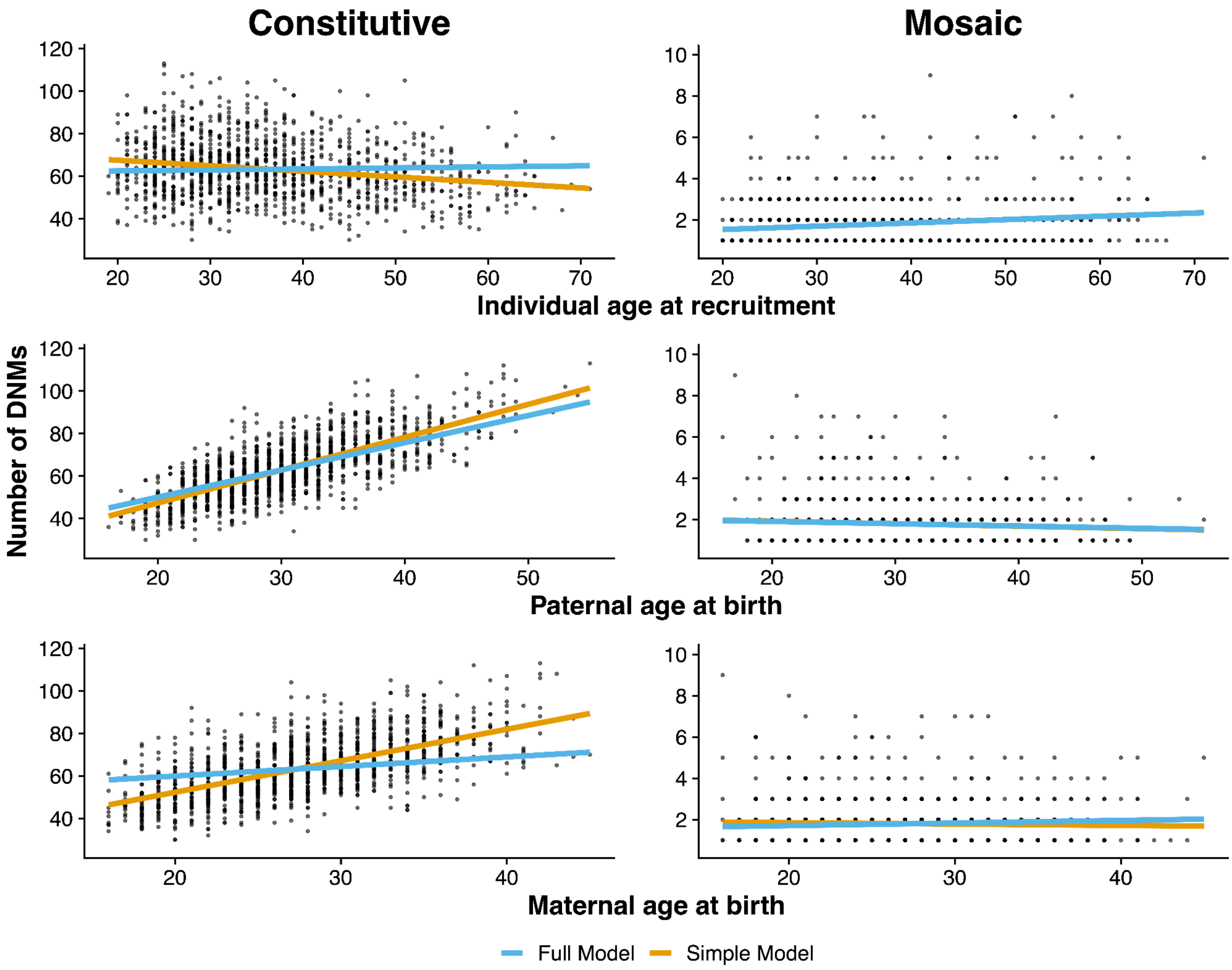

Figure S13: Number of constitutive and mosaic *de novo* variants in AoU.

Number of constitutive (left) and mosaic (right) *de novo* variants compared to proband age at recruitment (top), paternal age at birth (middle) or maternal age at birth (bottom) in AoU. Yellow = simple linear regression (no adjustment); Blue = full model (adjusted for ages of the other two members of the trio).

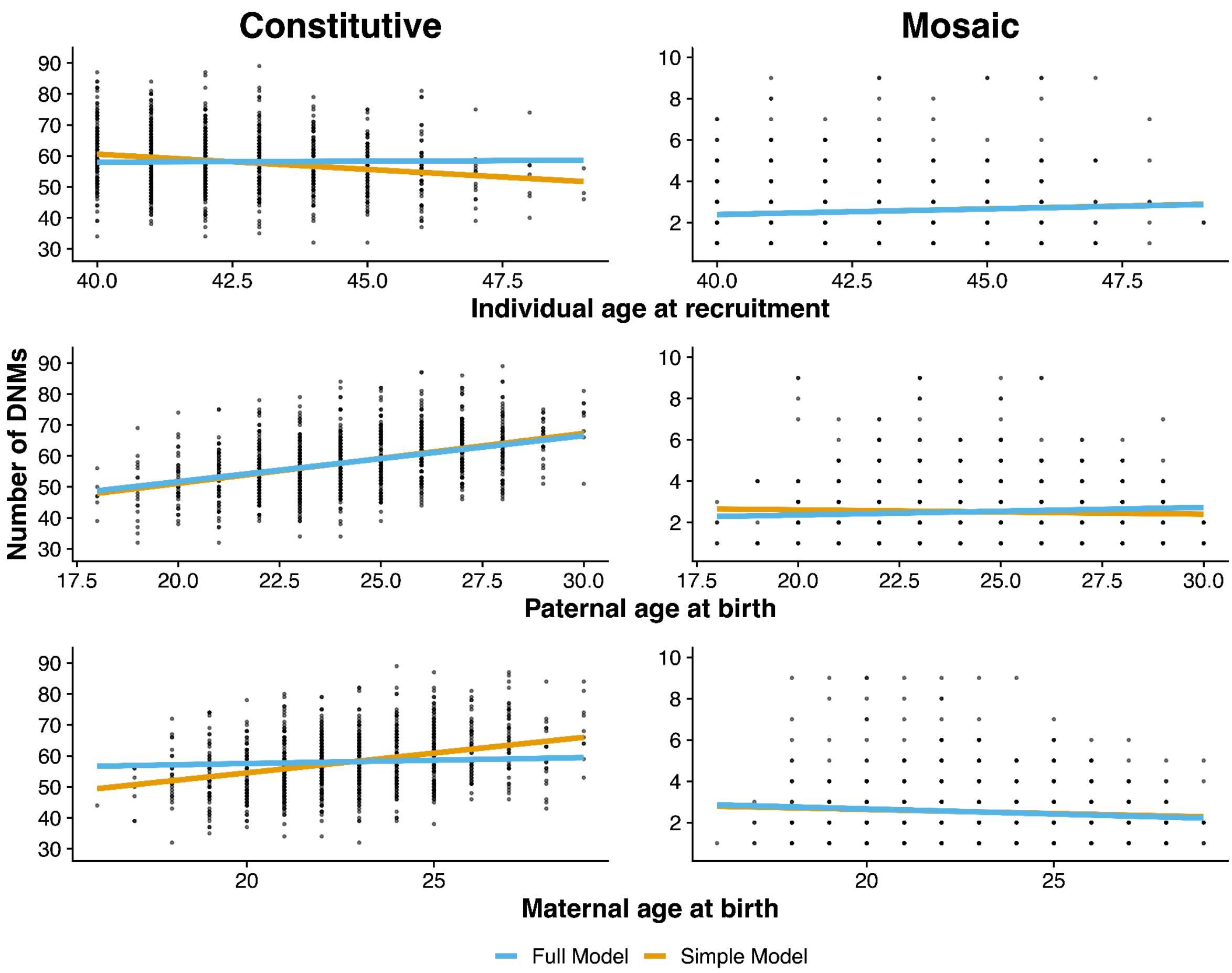

Figure S14: Number of constitutive and mosaic *de novo* variants in UKB.

Number of constitutive (left) and mosaic (right) *de novo* variants compared to proband age at recruitment (top), paternal age at birth (middle) or maternal age at birth (bottom) in UKB. Yellow = simple linear regression (no adjustment); Blue = full model (adjusted for ages of the other two members of the trio).

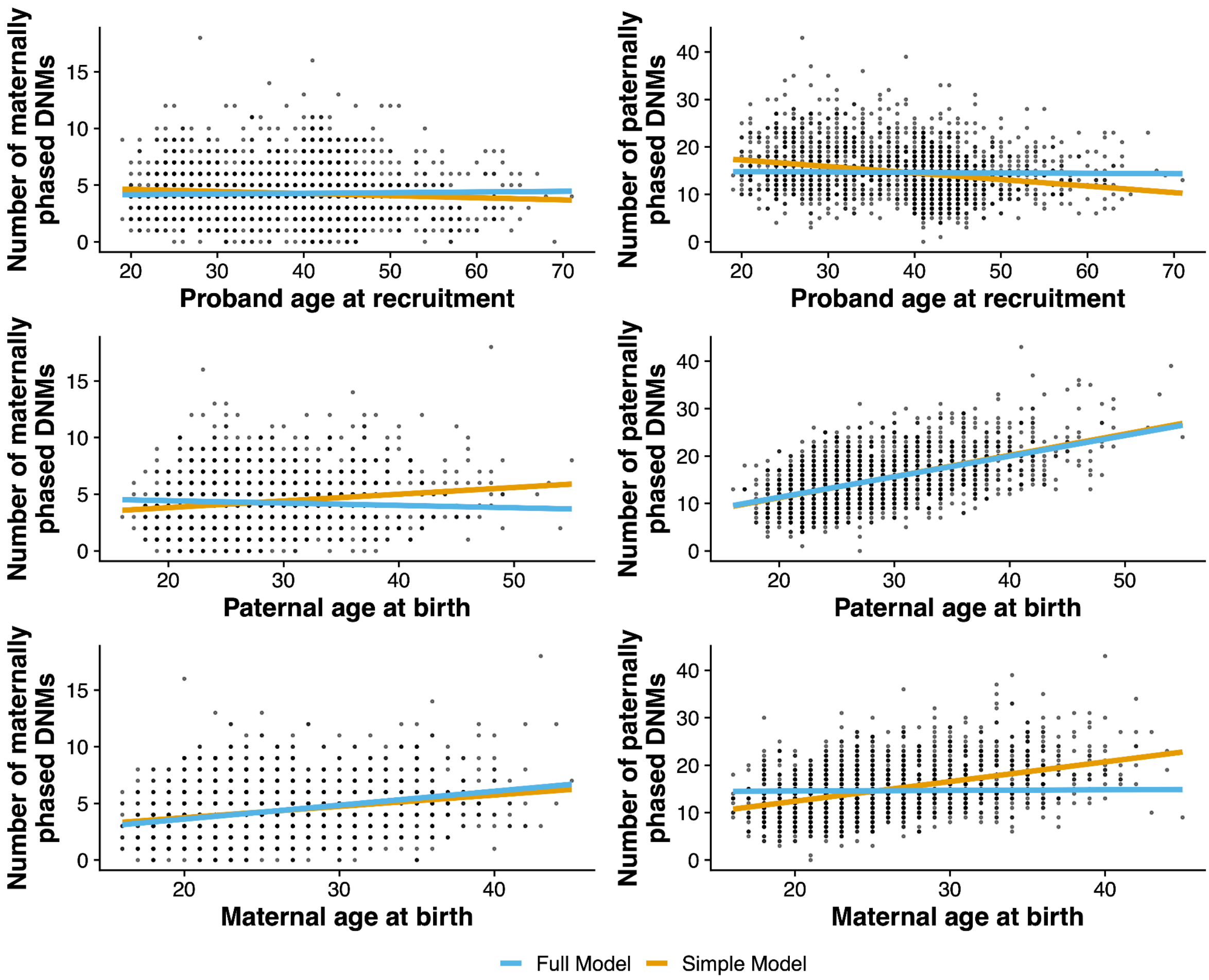

Figure S15: Number of paternally and maternally phased constitutive *de novo* variants.

Number of maternally phased (left) and paternally phased (right) *de novo* variants compared to proband age at recruitment (top), paternal age at birth (middle) or maternal age at birth (bottom) in AoU. Yellow = simple linear regression (no adjustment); Blue = full model (adjusted for ages of the other two members of the trio).

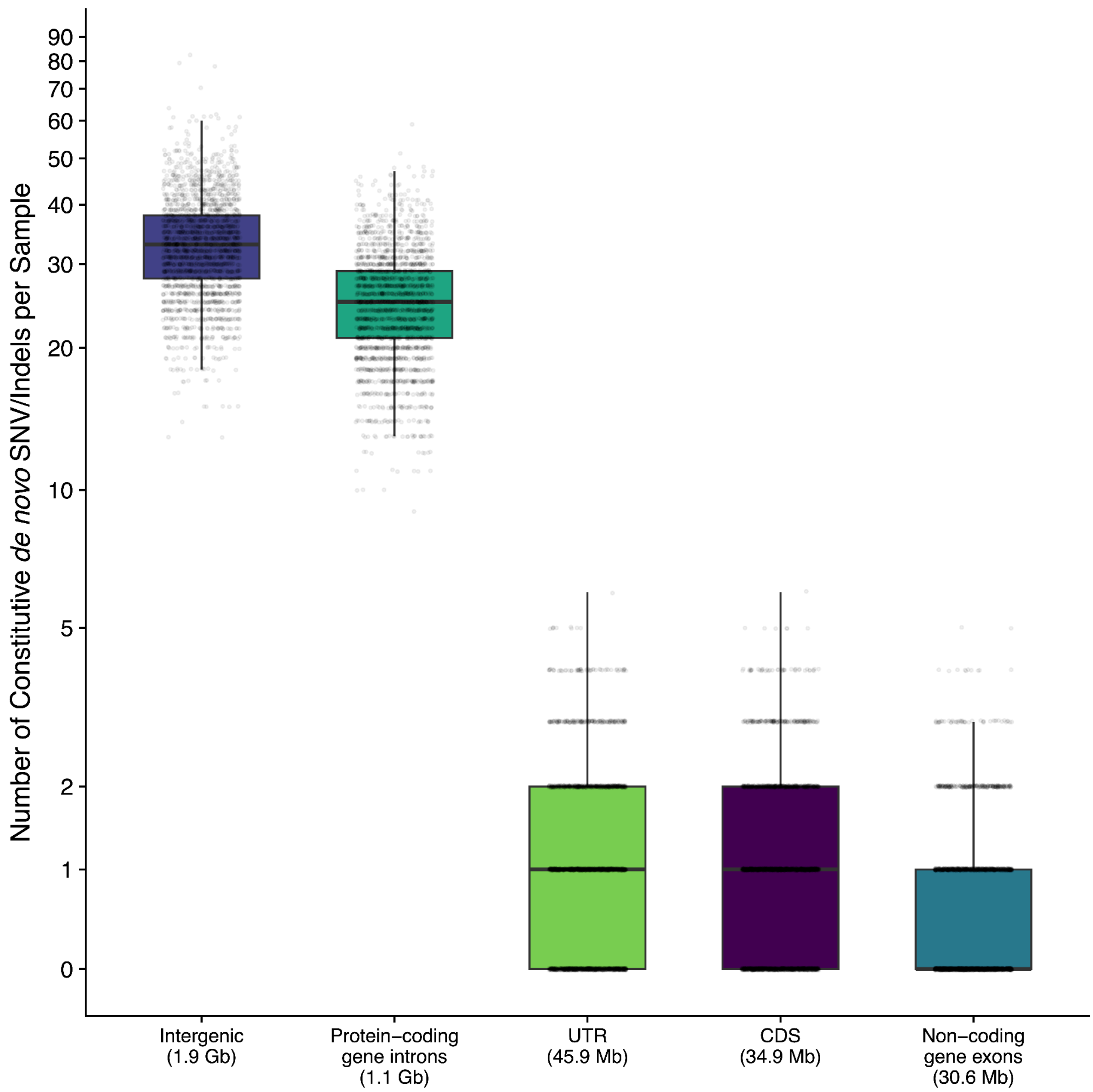

Figure S16: The number of constitutive *de novo* SNV/Indels within genomic regions.

Annotated regions were taken from GENCODE Primary transcripts from GENCODEv49. Untranslated regions (UTR) overlapping Protein-coding sequence (CDS) were excluded, intronic regions overlapping protein-coding gene exons were excluded, and non-coding gene exons overlapping protein-coding genes were excluded. GIAB low complexity regions, and segmental duplication regions were removed.

a

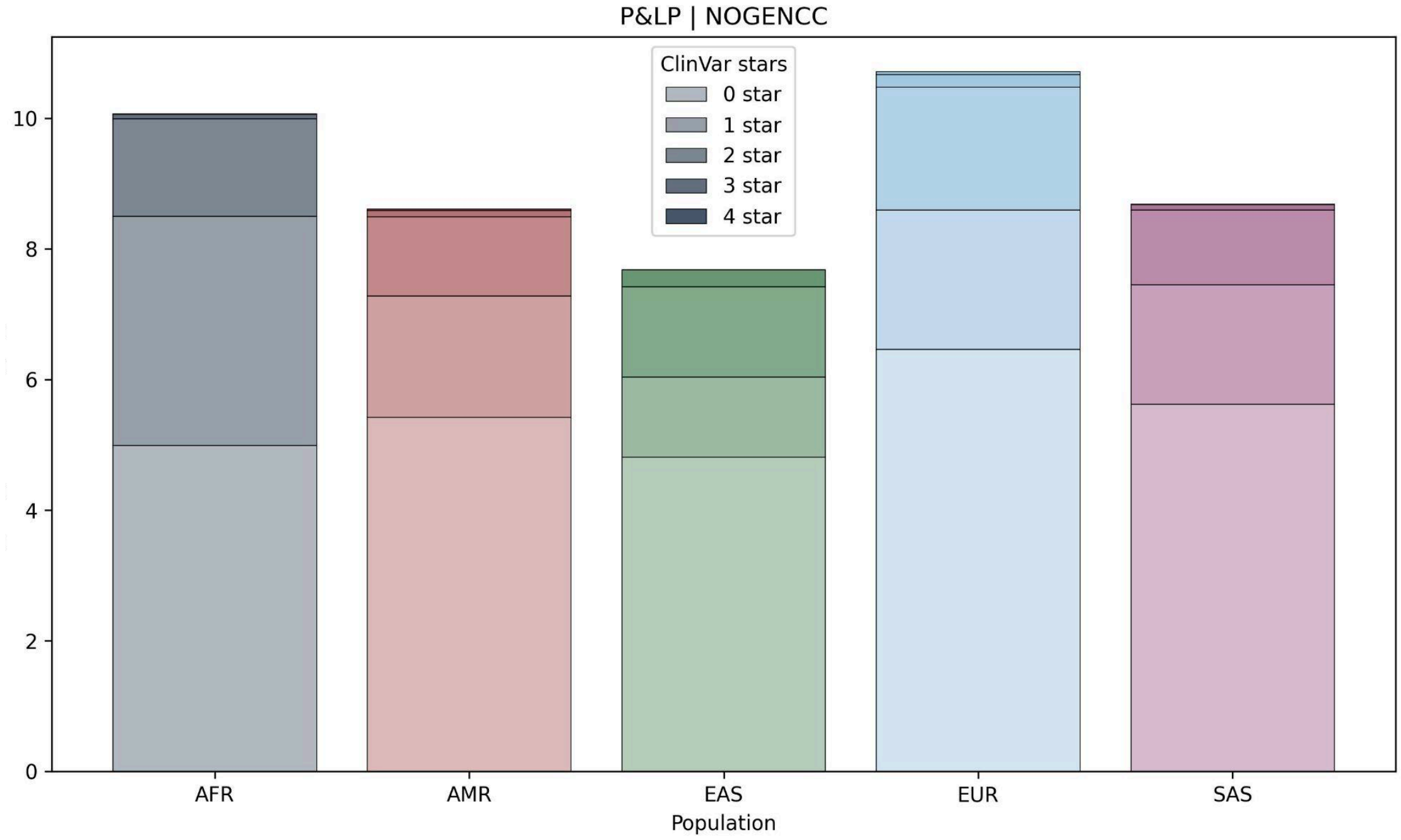

b

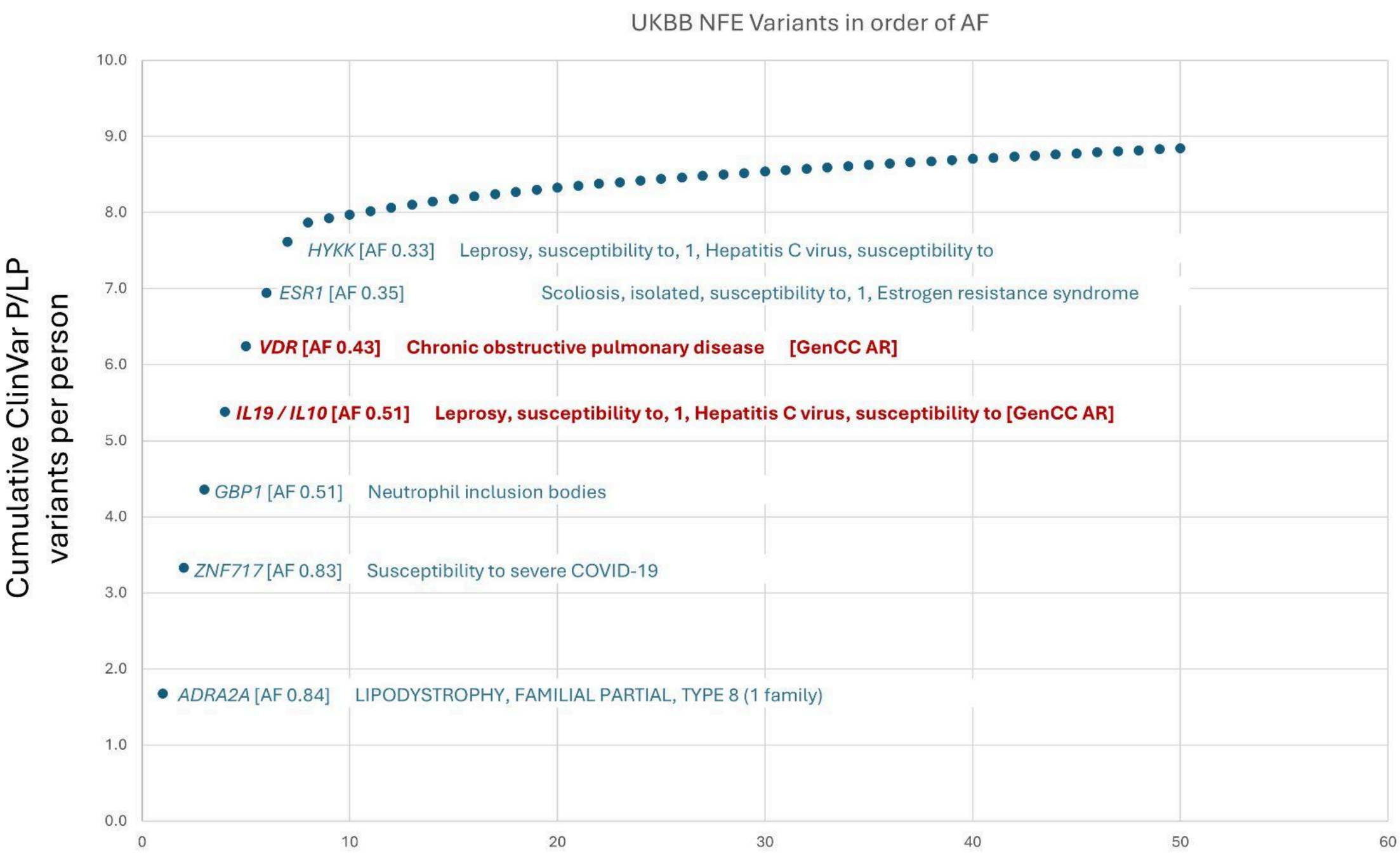

Figure S17: Initial Curation of ClinVar Variants.

a) Initial investigation of ClinVar P/LP variants identified 8-10 variants per genome; b) a small number of variants with extremely high allele frequencies contributed a disproportionate amount to the total estimate. Most of these variants (blue) were excluded by limiting the list to high confidence disease-associated genes in GenCC, however four variants with an AF >20% in one or more populations required manual exclusion.

P = pathogenic; LP = likely pathogenic; AF = allele frequency; GenCC = Gene Curation Coalition; AR = autosomal recessive.

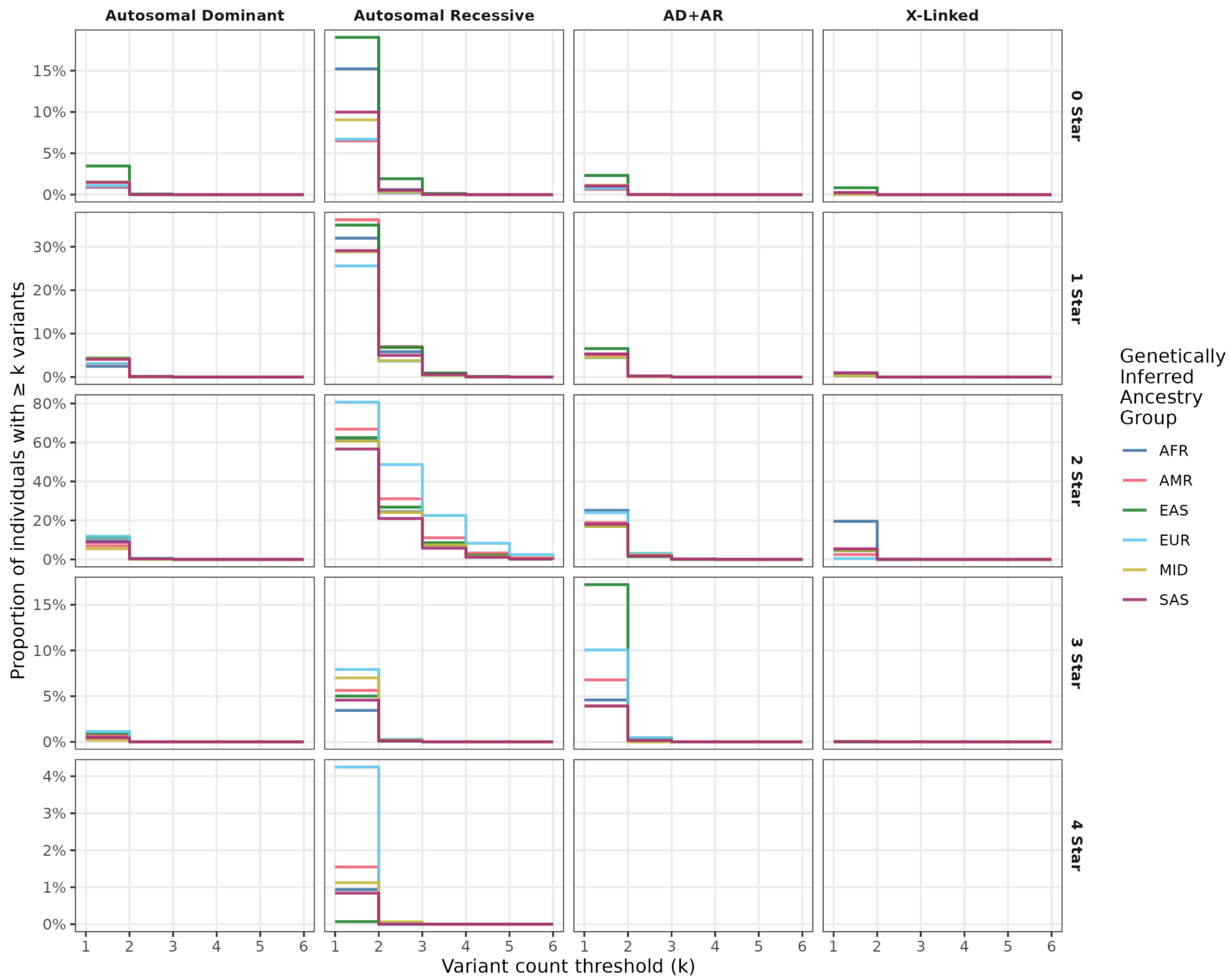

Figure S18: Percentage of individuals carrying pathogenic or likely pathogenic ClinVar variants.

Percentage of individuals across UKB and AoU with *k* or more ClinVar variants linked to GenCC-curated monogenic disease-associated genes, separated by inheritance type and star rating. For example, 80% of EUR have 1 or more 2* AR variants, with 50% having 2 or more. AFR = African, AMR = Admixed American, EAS = East Asian, EUR = European, MID = Middle-Eastern, SAS = South Asian. AD+AR = genes linked with both Autosomal Dominant (AD) and Autosomal (AR) Recessive conditions.

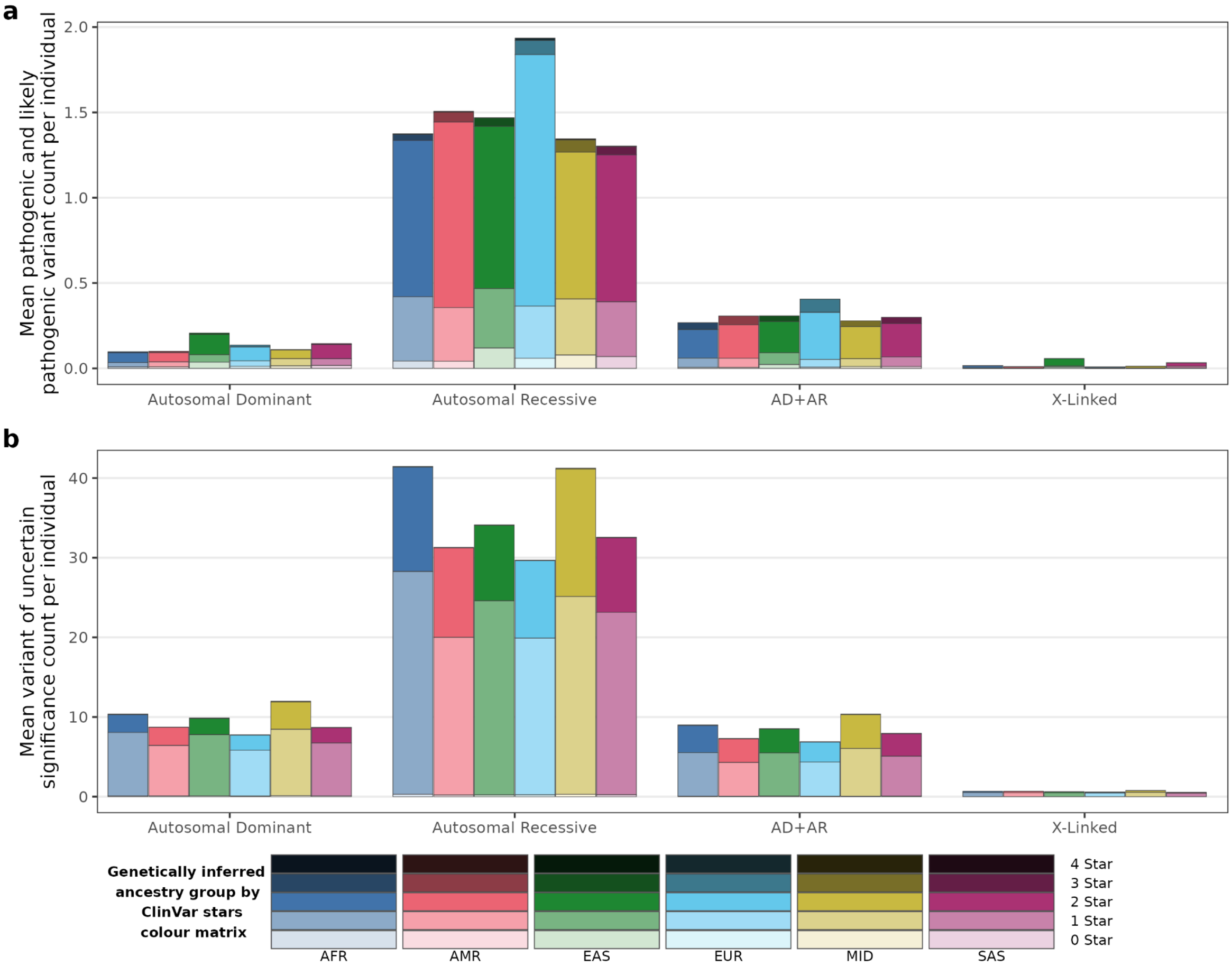

Figure S19: Mean number of MAF < 1% ClinVar variants in GenCC-curated monogenic disease-associated genes carried by individuals in AoU.

a) Number of ClinVar pathogenic/likely pathogenic variants per genome (MAF < 1%); b) number of ClinVar variants of uncertain significance per genome. Variants from AoU only. GenCC-curated disease genes were included if the genes were supported by definitive, strong or moderate levels of evidence without the associations being refuted or disputed (Supplementary Table S4). ClinVar star-rating relates to the level of review supporting the variant classification (4 = practice guideline; 3 = reviewed by expert panel; 2 = criteria provided, multiple submitters, no conflicts; 1 = criteria provided, single submitter; 0 = no assertion criteria). AD = autosomal dominant; AR = autosomal recessive; AD+AR = both AD and AR conditions.

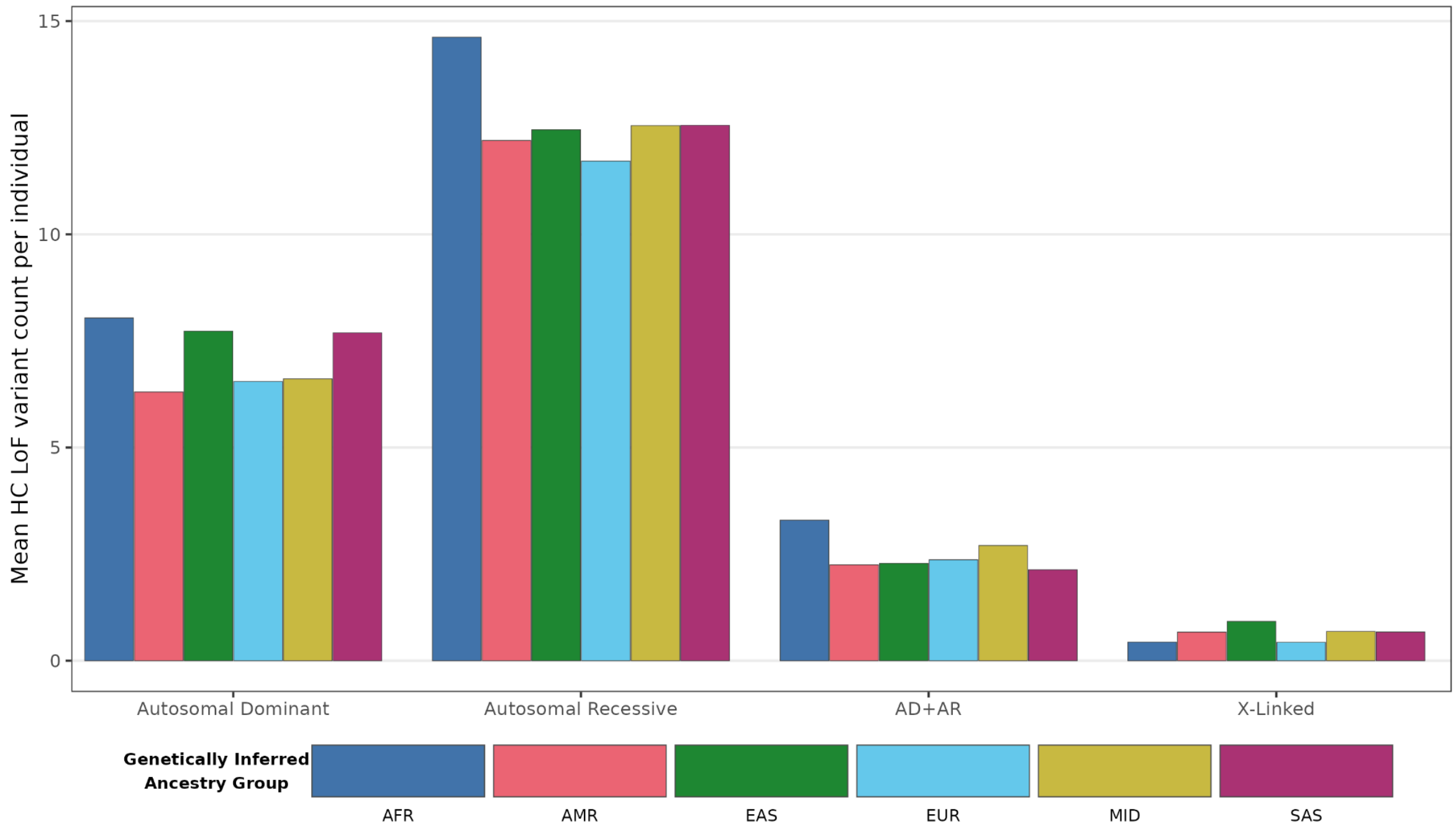

Figure S20: Mean number of high-confidence loss-of-function variants in GenCC-curated monogenic disease associated-genes carried by individuals.

AFR = African, AMR = Admixed American, EAS = East Asian, EUR = European, MID = Middle-Eastern, SAS = South Asian. AD + AR = genes linked with both Autosomal Dominant and Autosomal Recessive conditions.

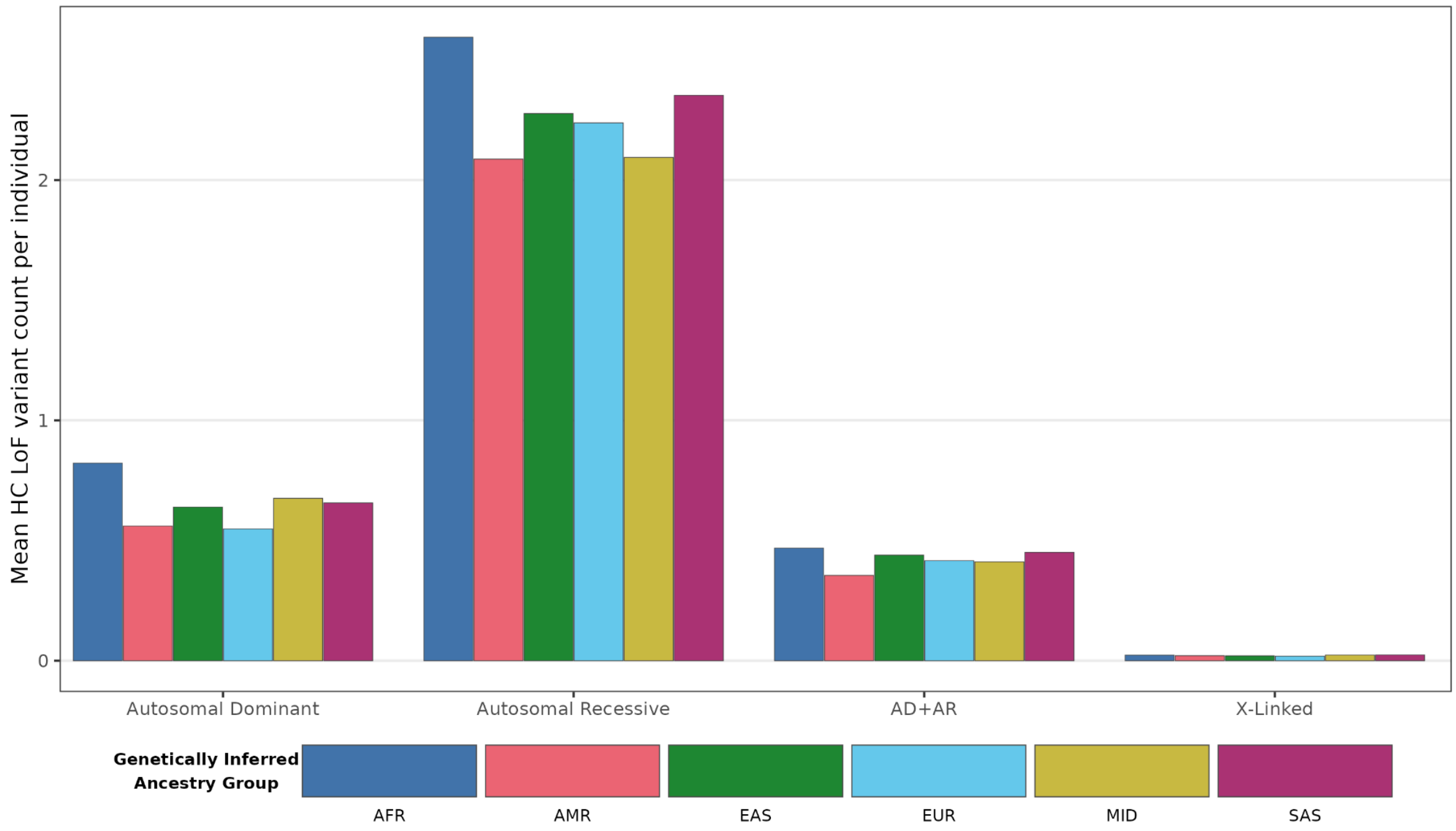

Figure S21: Mean number of MAF < 1% high-confidence loss-of-function variants in GenCC-curated monogenic disease associated-genes carried by individuals in AoU.

AFR = African, AMR = Admixed American, EAS = East Asian, EUR = European, MID = Middle-Eastern, SAS = South Asian. AD + AR = genes linked with both Autosomal Dominant and Autosomal Recessive conditions.

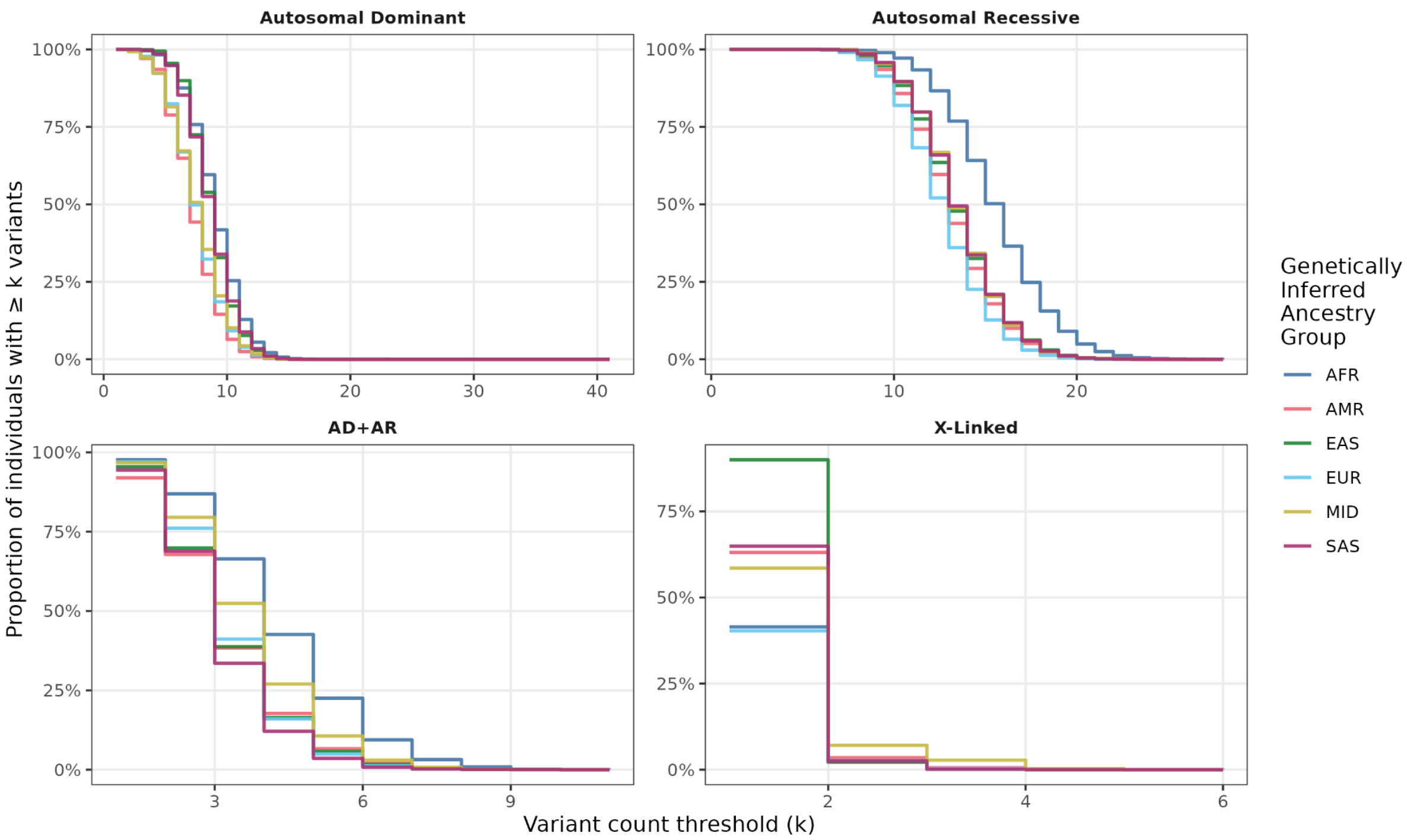

Figure S22: Percentage of individuals carrying high-confidence loss-of-function variants in GenCC genes.

Percentage of individuals across UKB and AoU with *k* or more HC LoF variants linked to GenCC-curated monogenic disease-associated genes, separated by inheritance type. AFR = African, AMR = Admixed American, EAS = East Asian, EUR = European, MID = Middle-Eastern, SAS = South Asian. AD + AR = genes linked with both Autosomal Dominant and Autosomal Recessive conditions.

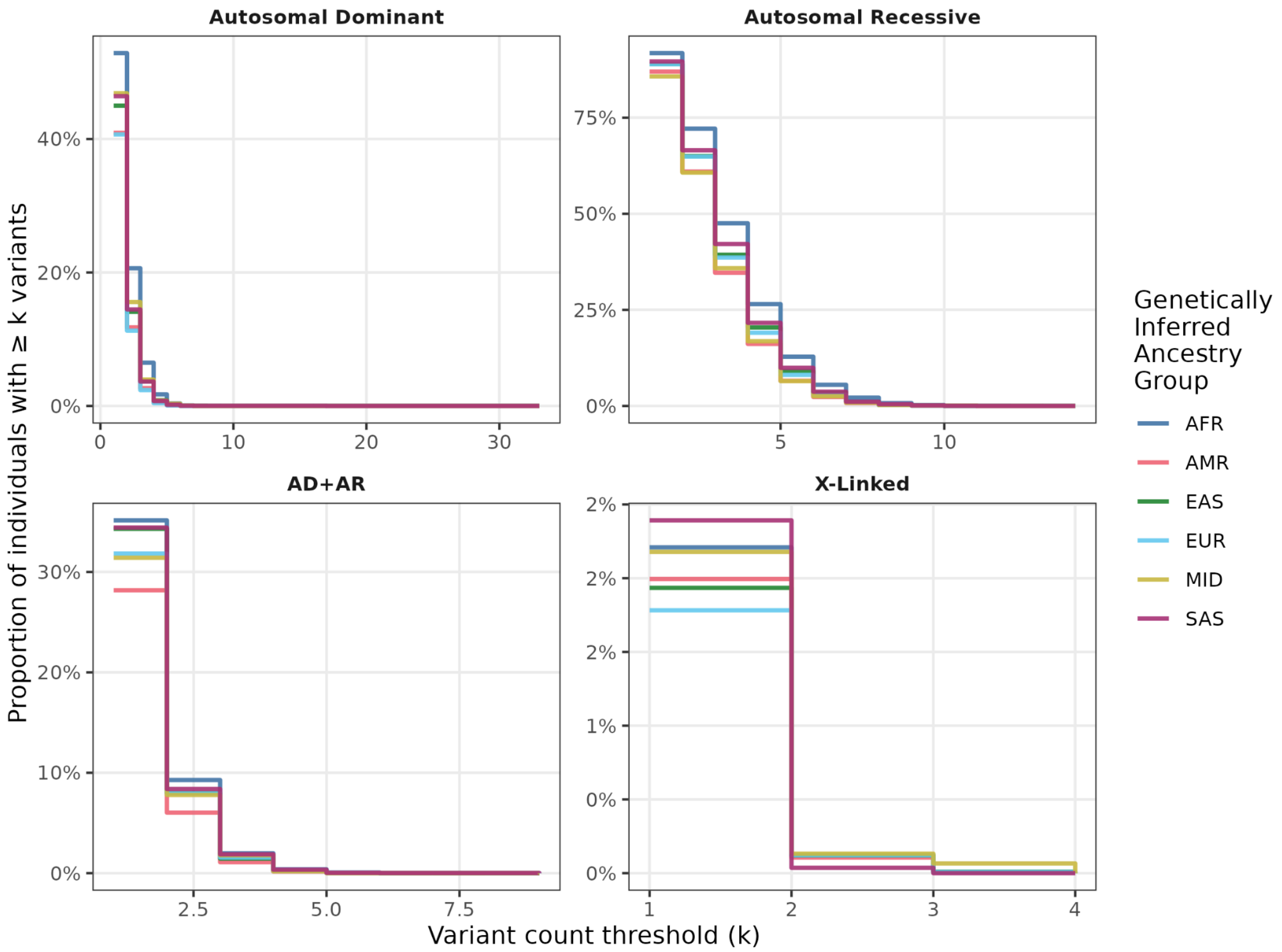

Figure S23: Percentage of individuals in AoU carrying MAF < 1% high-confidence loss-of-function variants in GenCC genes.

Percentage of individuals in AoU with *k* or more HC LoF variants linked to GenCC-curated monogenic disease-associated genes, separated by inheritance type. AFR = African, AMR = Admixed American, EAS = East Asian, EUR = European, MID = Middle-Eastern, SAS = South Asian. AD + AR = genes linked with both Autosomal Dominant and Autosomal Recessive conditions.

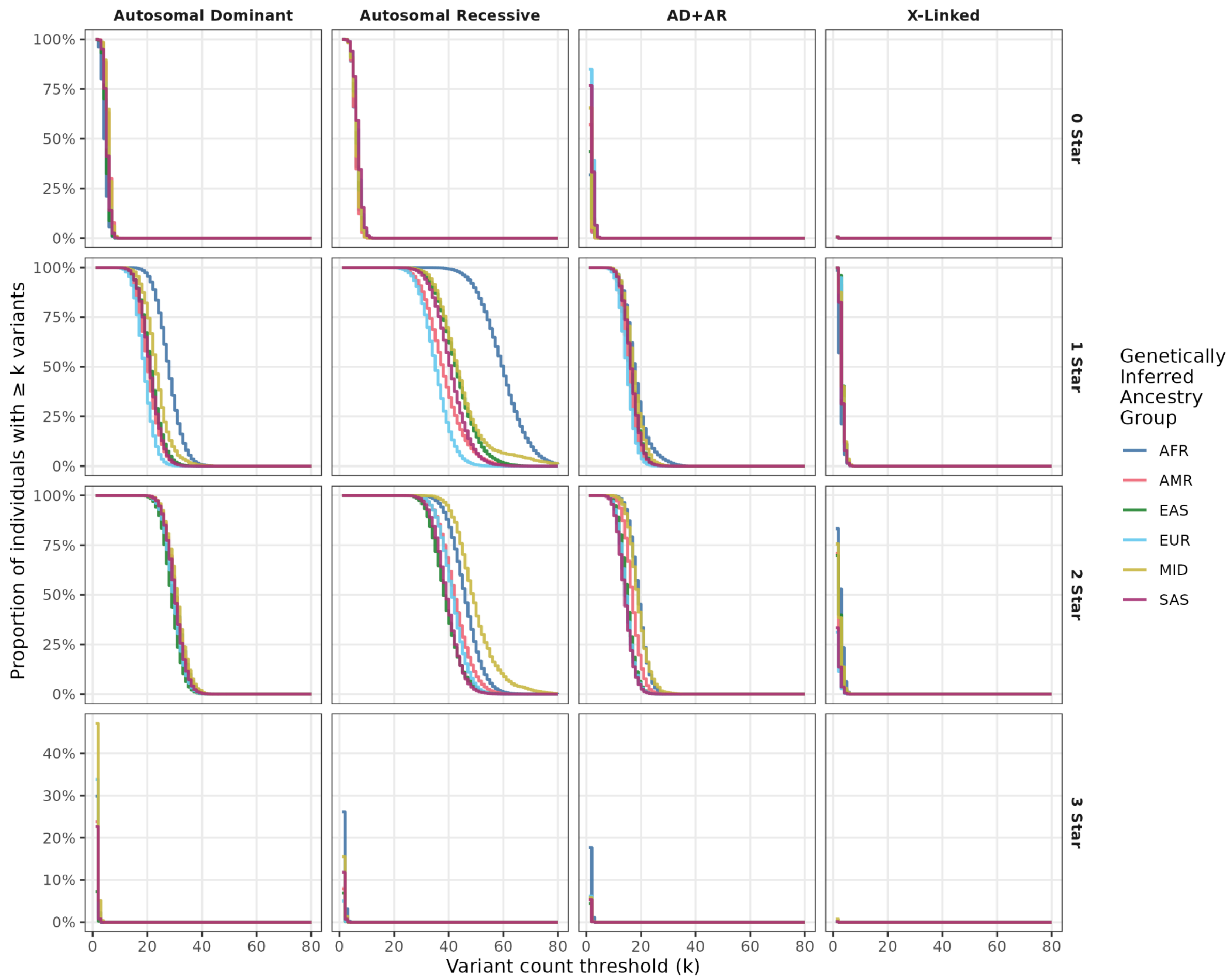

Figure S24: Percentage of individuals carrying ClinVar variants of uncertain significance.

Percentage of individuals across UKB and AoU with K or more ClinVar variants linked to GenCC-curated monogenic disease-associated genes, separated by inheritance type and star rating. For example, 100% of all groups have 1 or more 1* AR variants, with about 50% of SAS having 40 or more and 50% of AFR having 60 or more. AFR = African, AMR = Admixed American, EAS = East Asian, EUR = European, MID = Middle-Eastern, SAS = South Asian. AD + AR = genes linked with both Autosomal Dominant and Autosomal Recessive conditions.

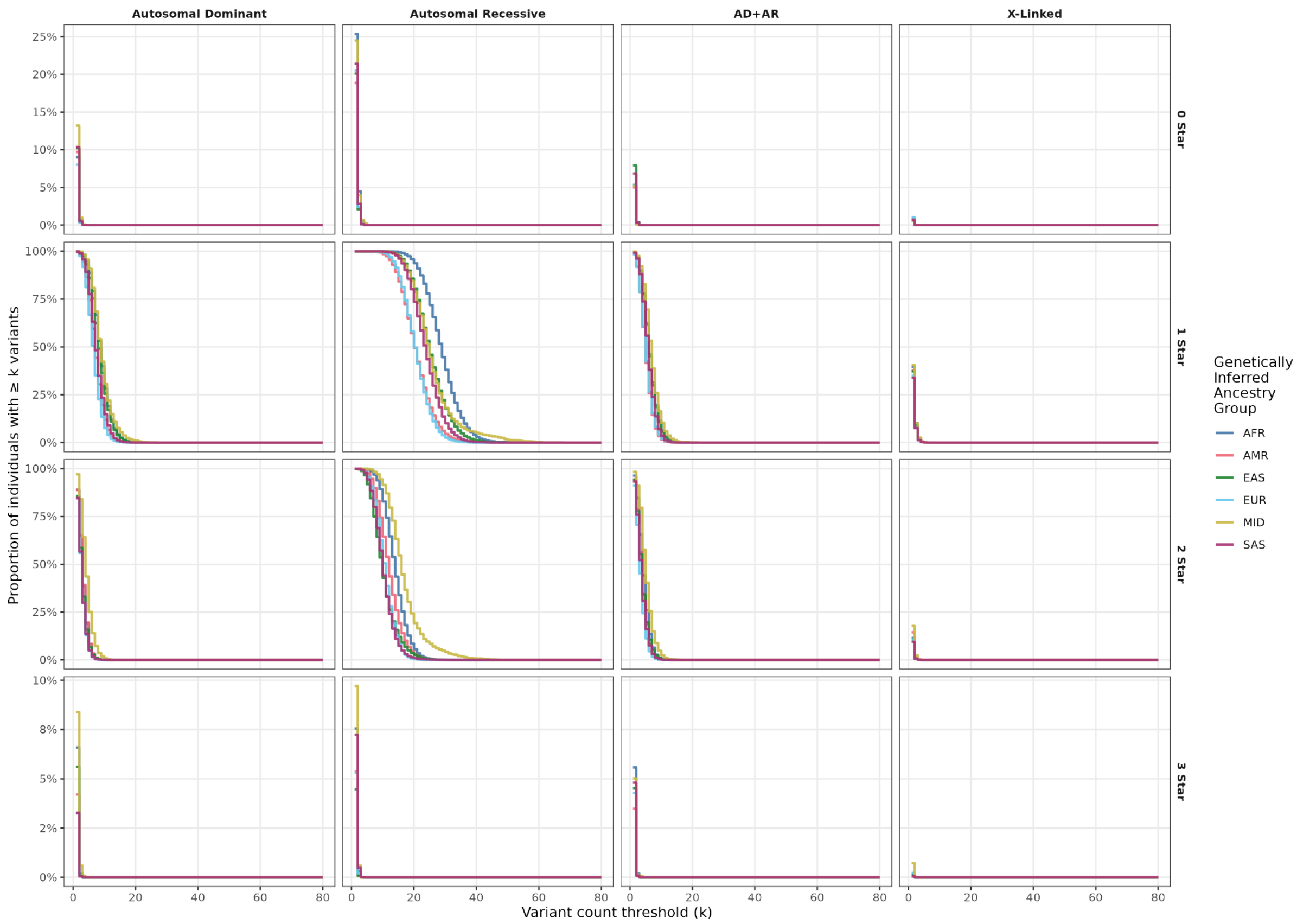

Figure S25: Percentage of individuals in AoU carrying MAF < 1% ClinVar variants of uncertain significance.

Percentage of individuals in AoU with K or more ClinVar variants linked to GenCC-curated monogenic disease-associated genes, separated by inheritance type and star rating. For example, 100% of all groups have 1 or more 1* AR variants, with about 50% of SAS having 40 or more and 50% of AFR having 60 or more. AFR = African, AMR = Admixed American, EAS = East Asian, EUR = European, MID = Middle-Eastern, SAS = South Asian. AD + AR = genes linked with both Autosomal Dominant and Autosomal Recessive conditions.

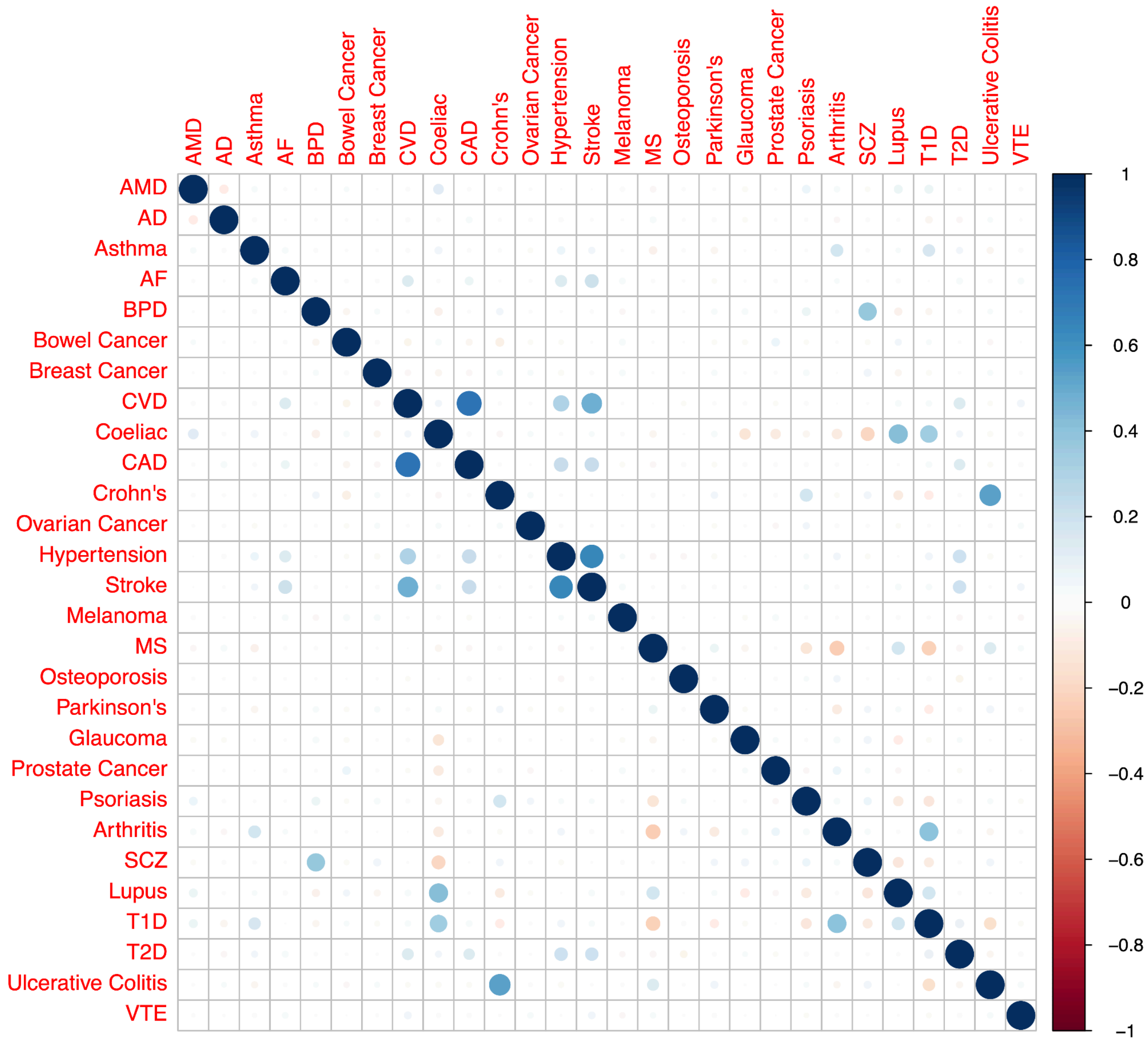

Figure S26: Correlation between PGSs in UKB.

Non-enhanced polygenic scores (PGSs) pre-calculated by Genomics Plc and available through the UKB-RAP from fields 26202 to 26289, limited to a subset of 28 disease-related PGSs. AD = Alzheimer's Disease; AF = Atrial fibrillation; AMD = Age-related Macular Degeneration; BPD = BiPolar Disorder; CAD = Coronary Artery Disease; CVD = CardioVascular Disease; MS = Multiple Sclerosis; SCZ = SChiZophrenia; T1D = Type 1 Diabetes; T2D = Type 2 Diabetes; UC = Ulcerative Colitis; VTE = Venous ThromboEmbolic disease.
